# Systematic review of the association between socioeconomic status and bladder cancer survival with hospital type, comorbidities, and treatment delay as mediators

**DOI:** 10.1002/bco2.65

**Published:** 2021-01-07

**Authors:** Beth Russell, Christel Häggström, Lars Holmberg, Fredrik Liedberg, Truls Gårdmark, Richard T Bryan, Pardeep Kumar, Mieke Van Hemelrijck

**Affiliations:** ^1^ Department of Translational Oncology and Urology Research School of Cancer and Pharmaceutical Sciences King's College London London UK; ^2^ Department of Surgical Sciences Uppsala University Uppsala Sweden; ^3^ Department of Public Health and Clinical Medicine Umeå University Umeå Sweden; ^4^ Department of Urology Skåne University Hospital Malmö Sweden; ^5^ Institution of Translational Medicine Lund University Malmö Sweden; ^6^ Department of Clinical Sciences Danderyd Hospital, Karolinska Institute Stockholm Sweden; ^7^ Institute of Cancer and Genomic Sciences The University of Birmingham Birmingham UK; ^8^ The Royal Marsden NHS Foundation Trust London UK

**Keywords:** bladder cancer, mediation, socioeconomic status, survival, systematic review

## Abstract

**Objectives:**

To review the current evidence on the relationship between three proposed mediators (comorbidities, hospital type, and treatment delays) for the relationship between socioeconomic status (SES) and bladder cancer survival.

**Materials and methods:**

Six different searches using OVID (Medline and Embase) were carried out to collate information available between the proposed mediators with both SES and survival in bladder cancer. This systematic review was conducted according to a pre‐defined protocol and in line with the Preferred Reporting Items for Systematic Reviews and Meta‐Analyses (PRISMA) guidelines.

**Results:**

A total of 49 studies were included in the review across the six searches (one appeared in two searches). There was a wealth of studies investigating the relationship between each of the proposed mediators with survival in bladder cancer patients. In general, a higher SES, lower comorbidities, and a larger hospital volume were all found to be associated with a decreased risk of death in bladder cancer patients. There was, however, a paucity of studies investigating the associations between these mediators and SES in bladder cancer patients.

**Conclusions:**

To gain a deeper understanding of the relationship between SES and survival identified in several observational studies, further investigations into the relationship between the proposed mediators and SES are warranted. Moreover, modifiable mediators, eg, treatment delay, highlight the importance of the standardization of clinical care across SES groups for all bladder cancer patients.

## 1 INTRODUCTION

The survival of bladder cancer patients is dependent on many factors such as clinical variables, gender, diagnostic delay, geographical region, access to care, comorbidity, and risk factors such as smoking and occupational exposure.^1^ Many of these individual factors are associated with socioeconomic status (SES). Disparities in cancer incidence and survival have been frequently observed among different socioeconomic groups for several types of cancer including bladder, stomach, liver, lips–mouth–pharynx, and lung.^2^, ^3^

In bladder cancer, the link between SES and survival has been studied previously and patients with lower SES have been shown to have a decreased 5‐year survival,^4^ overall survival,^5^ and higher relative risk of death.^6^ Null finding has also been observed for the risk of bladder‐cancer‐specific mortality.^5^ Around 75% of all bladder cancer patients have non‐muscle‐invasive bladder cancer (NMIBC) with the remaining 25% being diagnosed with muscle‐invasive bladder cancer (MIBC). The main treatment choice for patients with NMIBC is usually transurethral resection of the bladder tumor (TURBT), Bacillus Calmette‐Guerin (BCG) therapy or chemotherapy.^7^ For non‐metastatic MIBC, the main treatment choices are radical cystectomy, chemotherapy or radiotherapy.^8^

The exact mechanism behind the association between SES and survival is complex and unknown; however, a review by Quaglia et al. postulated that the link could be explained by factors relating to three main groups: diagnosis, treatment modalities, and patient characteristics.^9^ Despite this evidence, there remains paucity in detailed studies and comprehensive clinical investigations to elucidate the underlying mechanisms behind this association. SES is a largely unmodifiable factor; therefore, identifying potential mediators of the association between SES and survival could be used as a foundation for future interventions or recommendations to reduce the SES disparity seen in cancer survival.

Previously, using data from a cohort of Swedish bladder cancer patients, we found that Charlson Comorbidity Index (CCI), hospital type, and treatment delays mediated the association between SES and risk of death.^10^ On the basis of these results, the aim of this systematic review is to collate information from existing literature about the potential mediators (hospital type, comorbidities, and treatment delay) for the association between SES and survival in bladder cancer patients.

## 2 MATERIALS AND METHODS

**FIGURE 1:**
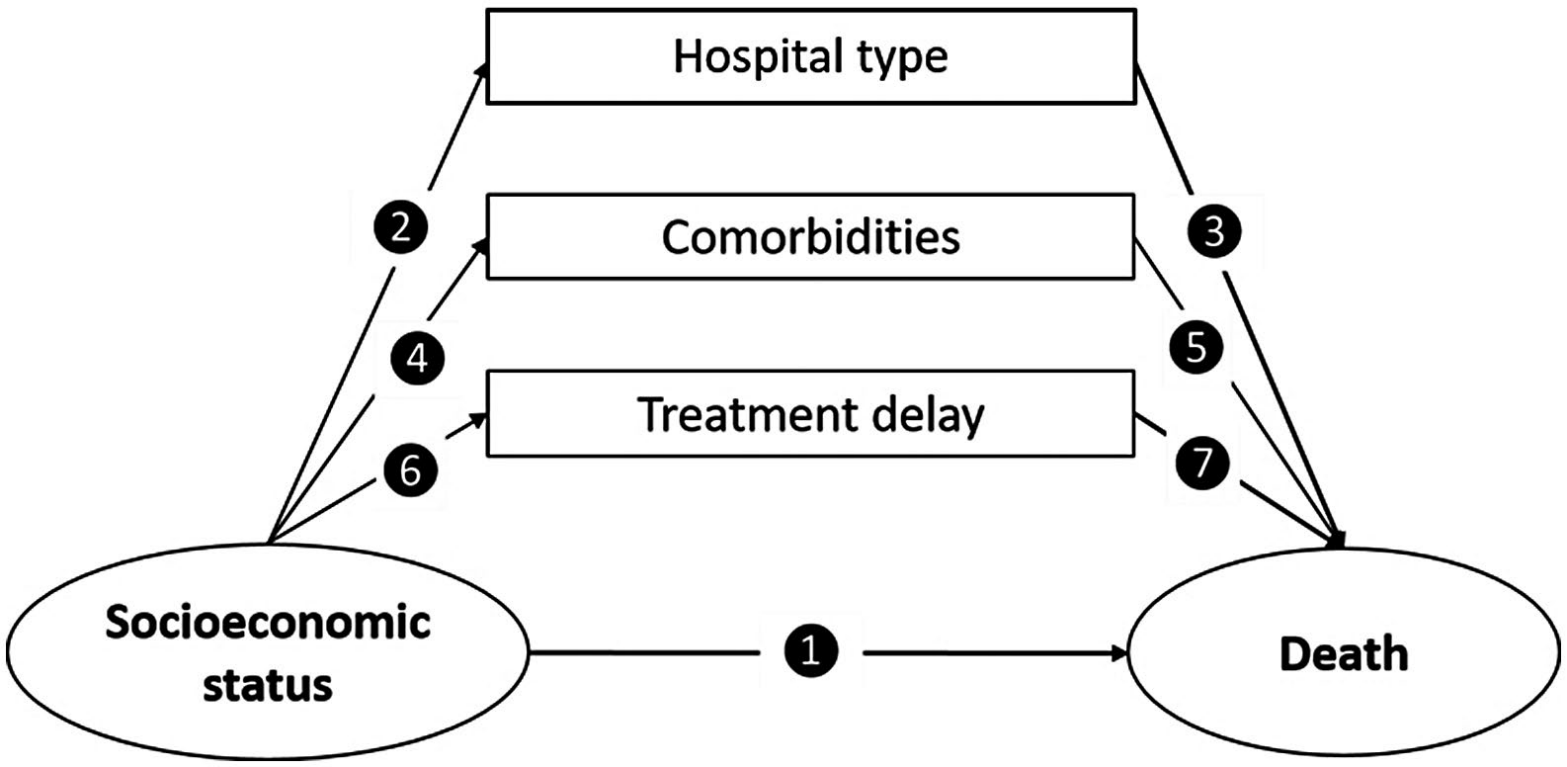
Directed acyclic graph with each number depicting a different association investigated. Socioeconomic status is the exposure variable, death is the outcome variable, while hospital type, comorbidities, and treatment delay are the potential mediators

Using similar methods as those described by Shanmugalingam et al,^11^ six separate searches were performed to investigate the relationship of each potential mediator (hospital type, comorbidities, and treatment delay) with both SES and survival in bladder cancer patients. The full protocol is outlined in the Appendix. Figure 1 demonstrates the directed acyclic graph with each arrow representing a search carried out. The six searches were as follows: (1) SES and survival, (2) SES and hospital type, (3) Hospital type and survival, (4) SES and comorbidities, (5) comorbidities and survival, (6) SES and treatment delay. Number 7 in Figure 1 (the association between treatment delay and survival), however, was not carried out as the results are presented elsewhere.^12^

### 2.1 Search strategies

The online database Ovid Gateway was used to search both Embase and Medline for relevant studies (for which the search items are explained in detail in the appendix). Searches were performed to include only articles in English, with human subjects, and published from the year 2000 onwards. Searches were performed in November 2019.

Articles were included if: they were specific to bladder cancer, they were not conference abstracts or commentaries and the full text was available. Studies were excluded if they did not investigate the relevant exposure and outcome variables for each mediator and/or were deemed of low quality after quality assessment (as explained below). Reviews were included to allow for the inclusion of as much information as possible including data from studies that might not have been captured within the current search strategy. When systematic reviews were already conducted according to the PRISMA guidelines, these were considered to overwrite individual studies included in the systematic review and the results were presented as a whole. Initially, titles were screened for relevance, then abstracts and full texts were subsequently screened. Data extraction was performed on a per mediator basis in which the year, country of study, number of patients, method for assessing comorbidities or socioeconomic status, and summary measures of results (e.g. survival proportions, odds ratios or hazard ratios) were recorded in separate tables. All screening was performed by BR and MVH and data extraction by BR. All studies were described and compared in a narrative manner with no quantitative analyses taking place.

This review was conducted in accordance with the Preferred Reporting Items for Systematic Reviews and Meta‐Analyses (PRISMA) guidelines.^13^ The quality of the studies was assessed using Risk of Bias in Non‐Randomized Studies – Interventions (ROBINS‐I) for all observational studies with death as the outcome.^14^ The systematic reviews were assessed using A Measurement Tool to Assess Systematic Reviews 2 (AMSTAR 2),^15^ while the narrative reviews were assessed using the Scale for the quality Assessment of Narrative Review Articles (SANRA).^16^

## 3 RESULTS

**FIGURE 2:**
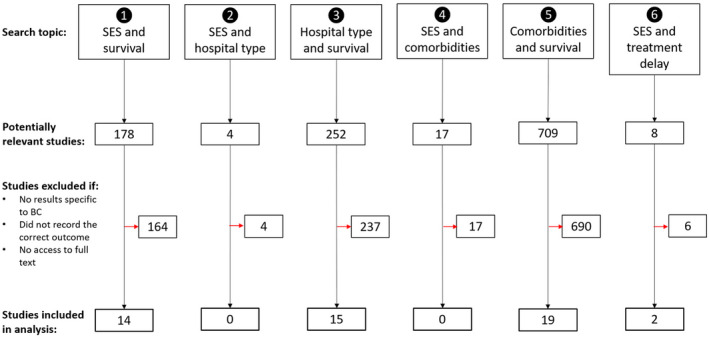
Study selection process. BC, bladder cancer; SES, socioeconomic status

A total of 1168 studies were initially extracted from the searches. A summary of the frequency of studies identified and subsequently included in the analysis for each search is depicted in Figure 2. No studies were identified for inclusion for the associations between SES and both comorbidities and hospital type (arrows 2 and 4, Figure 2).

### 3.1 SES and survival

After screening the 178 studies identified in the search, 14 studies were deemed suitable for inclusion when investigating the association between SES and survival (both overall and bladder cancer‐specific) in bladder cancer patients^5^, ^28^ (Table 1). The main reason for the exclusion of studies was the investigation of post‐operative complications rather than survival specifically. Included in the analysis were 2 reviews and 12 cohort studies.

**TABLE 1:** Studies identified relating to socioeconomic status and survival

Authors	Reference	Title	Year	Country of study	Study type	Patients	SES indicator	Summary of results	Association
Quaglia et al.	(9)	Socio‐economic inequalities: A review of methodological issues and relationships with cancer survival	2013	Italy	Review	N/A	N/A	Lower SES resulted in reduced survival among all cancers, with the relative risks being particularly high in bladder cancer patients	+
Shackley and Clarke	(17)	Impact of socioeconomic status on bladder cancer outcome	2005	UK	Review	N/A	N/A	Included results from three studies looking at survival. All three studies found an increased risk of overall death among those in a lower SES group	+
Lara et al.	(18)	Determinants of survival in adolescents and young adults with urothelial bladder cancer: results from the California Cancer Registry	2016	USA	Observational study	104 974 newly diagnosed BC patients (1688 of which were < 40 years of age)	Neighborhood SES	(In those aged 15‐39) Non‐Hispanic African Americans with low SES were found to have an increased risk of cancer‐specific death (HR = 7.10, *P* < .001) when compared to non‐Hispanic Whites with the same SES level. In contrast, Non‐Hispanic African Americans with high SES were found to have a similar risk of cancer‐specific death to non‐Hispanic Whites in the same SES group (HR = 1.22, *P* = .79). This relationship was not observed in older patients	+
Eberle et al.	(19)	Socioeconomic inequalities in cancer incidence and mortality ‐ A spatial analysis in Bremen, Germany	2010	Germany	Observational study	27 430 newly diagnosed cancer patients of which 949 were BC	Assignment of town district to existing social class index	There was no obvious correlation between SES and mortality in bladder cancer patients	Null
Sloggett et al.	(20)	The association of cancer survival with four socioeconomic indicators: A longitudinal study of the older population of England and Wales 1981‐2000	2007	UK	Observational study	First primary cancer diagnosis aged 45 years or above (Total n = 26 273, BC patients n = 1407)	Carstairs, Car Access, Tenure, and Social Class	In BC patients, three of the four models concluded an increased hazard of excess mortality (Carstairs, Car Access, and Tenure models) with the largest of these being the Car Access model (HR = 2.30, 95% CI: 1.52‐3.48)	+
Klapheke et al.	(21)	Sociodemographic disparities in chemotherapy treatment and impact on survival among patients with metastatic bladder cancer	2018	USA	Observational study	Metastatic BC patients (n = 3667)	Neighborhood SES	Metastatic BC patients in the middle, lower‐middle and lowest SES group all had an increased risk of overall death when compared to those in the highest SES group (HR = 1.3, 95% CI: 1.1‐1.4; HR = 1.1, 95% CI: 1.0‐1.2; HR = 1.2, 95% CI: 1.0‐1.3 respectively)	+
Chien et al.	(22)	Patterns of age‐specific socioeconomic inequalities in net survival for common cancers in Taiwan, a country with universal health coverage	2018	Taiwan	Observational study	Patients aged 15‐94 with invasive cancers (Total n = 724 992, BC patients n = 19 033)	Insurable monthly income	There was a significant difference in net survival between the high and low (5.03%, 95% CI:1.82%‐8.24%), and medium and low SES groups (3.69%, 95% CI:2.01%‐5.37%) in the BC patients. When stratified by age, this association remained for those aged 15‐64	+
Begum et al.	(5)	Socio‐economic deprivation and survival in bladder cancer	2004	UK	Observational study	Patients newly diagnosed with urothelial cancer (n = 1537)	Enumeration district	Patients in the more affluent group had an increased overall survival when compared to those in the least affluent group (5‐year survival rates of 63%–67% for quintiles first and second versus 56% for quintiles fourth and fifth [*P* = .02]). No difference in BC‐specific survival was found	+
Moran et al.	(27)	Bladder cancer: worse survival in women from deprived areas	2004	UK	Observational study	Patients newly diagnosed with urothelial cancer (n = 1190)	Townsend score of the enumeration district (ED)	In women, the 6‐month survival was 73.5% for the less deprived compared with 52.3% for the more deprived (*P* < .05). Those from the more deprived areas were more likely to present with advanced disease (37.2%) compared to those from less deprived areas (8.8%) (*P* < .01)	+
Coleman et al.	(28)	Trends and socioeconomic inequalities in cancer survival in England and Wales up to 2001	2004	UK	Observational study	Patients diagnosed with 20 most common cancers (Total n = 2 207 865, BC patients n = 141 531)	Carstairs (patients diagnosed 1986–95) Indices of multiple deprivation (IMD) (patients diagnosed 1996–99)	5‐year survival proportions were 66% for men and 56% for women. The deprivation gap in 5‐year survival was −5.7% in men (95% CI: −8.2 to 3.1) and −5.8% in women (95% CI: −9.5 to 2.0)	+
Belot et al.	(23)	Describing the association between socioeconomic inequalities and cancer survival: Methodological guidelines and illustration with population‐based data	2018	France	Observational study	Cancer cases diagnosed 1997‐2010 (Total n = 67 691, BC patients n = 2148 men, n = 532 women)	European Deprivation Index (EDI)	The difference in 5‐year age‐standardized net survival between Q1 and Q5 (for EDI) was around 6% for women and almost 7% for men with bladder cancer. Excess HR for men 1.03 (95% CI: 1‐1.05), and for women 1.01 (95% CI: 0.98‐1.04)	+
Syriopoulou et al.	(24)	Estimating the impact of a cancer diagnosis on life expectancy by socio‐economic group for a range of cancer types in England	2017	UK	Observational study	Cancer cases diagnosed 1998‐2013 (Total n = 2 512 745, BC patients n = 100 821 men, n = 39 021 women)	Income domain of the index of multiple deprivation (IMD)	The percentage of life lost in the most deprived compared to the least deprived was 48.26% and 45.42% in men and 60.89% and 51.17% in women. Bladder had one of the lowest total life years lost across the varying groups’ deprivation	Not stated
Sundquist et al.	(25)	Neighborhood deprivation and mortality in individuals with cancer: A multilevel analysis from Sweden	2012	Sweden	Observational study	Cancer cases diagnosed 1990‐2004 (Total n = 400 169, BC patients n = 14 946 men, n = 4647 women)	Neighborhood deprivation	The odds of mortality in patients in the most deprived quartile was OR = 1.16 (95% CI:1.04–1.29) in men and OR = 1.36 (95% CI:1.12–1.66) in women	+
Shack et al.	(26)	Socioeconomic inequalities in cancer survival in Scotland 1986‐2000	2007	UK	Observational study	Patients diagnosed with 18 most common cancers in Scotland (Total n = 357 658, BC patients n = 3081 men, n = 1451 women)	Carstairs (patients diagnosed 1986–95) Indices of multiple deprivation (IMD) (patients diagnosed 1996–99)	5‐year survival proportions were 62.6% for men and 51.8% for women. The deprivation gap in 5‐year survival was −6.7% in men (95% CI: −12.6 to 0.8) and −7.3% in women (95% CI: −15.4‐0.7)	+

Note

BC, bladder cancer; HR, hazard ratio; SES, socioeconomic status; 95% CI – 95% confidence interval. “+” denotes positive association (ie higher SES associated with increased survival/lower SES associated with higher risk of death), “null” denotes a null association

Seven of the studies included both NMIBC and MIBC patients within their analyses and four of these concluded an association between a lower SES and reduced overall survival^5^, ^18^, ^20^, ^23^, ^28^ (Table 1). The two studies which did not observe this result were those of Eberle et al and Syriopoulou, although the latter only presented raw data with no level of significance reported for any results.^19^, ^24^ Four of the studies included MIBC only^22^, ^25^, ^26^, ^27^ and one study included metastatic MIBC patients only.^21^ All five concluded an association between a lower SES and reduced overall survival. None of the studies included any of the proposed mediators (comorbidities, hospital type or treatment delay) within their analyses.

### 3.2 Hospital type and survival

Fifteen studies investigating the association between hospital type and the survival of bladder cancer patients were identified^29^, ^30^, ^31^, ^32^, ^33^, ^34^, ^35^, ^36^, ^37^, ^38^, ^39^, ^40^, ^41^, ^42^, ^43^ (Table 2). Two studies were reviews, the remaining thirteen were cohort studies. All of the studies defined the “type” of hospital based on the volume of bladder cancer patients treated (namely the number of radical cystectomies per year). The studies by Bajaj et al^33^ and Scarberry et al^35^ additionally investigated whether the hospital was an academic center or not. The review by Nuttal et al^29^ noted that the approach to defining hospital volume was heterogeneous across studies, with some using the annual number of radical cystectomies and others using the number of radical cystectomies within the study period. All studies included patients with MIBC either undergoing radical cystectomy or curative radiotherapy (including chemoradiation therapy); the studies by Birkmeyer et al^32^ and Hounsome et al^36^ additionally included NMIBC patients.

**TABLE 2:** Studies identified relating to hospital type and survival

Authors	Reference	Title	Year	Country	Type of study	Measurement of hospital type	Patients	Summary of results	Association
Bajaj et al.	(33)	The Impact of Academic Facility Type and Case Volume on Survival in Patients Undergoing Curative Radiation Therapy for Muscle‐Invasive Bladder Cancer	2018	USA	Observational study	Academic vs. non‐academic and case volume	cT2 to cT4 N0 M0 transitional cell MIBC patients (2004‐2013) Treated with either curative RT or concurrent chemoradiation therapy	Patients treated at an academic hospital were not associated with improved overall survival when compared to those treated at a non‐academic hospital (HR, 0.94; 95% CI 0.84‐1.06)	Null
Nuttal et al.	(29)	A systematic review and critique of the literature relating hospital or surgeon volume to health outcomes for 3 urological cancer procedures.	2004	N/A	Review	Hospital volume (e.g. cases performed annually, or cases performed in the study period)	Patients who have had an RC. Number of patients not stated.	Out of four studies, one found a significant association between reduced mortality after RC with increasing hospital volume. Two studies found no significant difference in mortality between hospitals of varying volumes. The other study did not measure mortality after RC	Mixed
Goossens‐Laan et al.	(31)	Survival after treatment for carcinoma invading bladder muscle: a Dutch population‐based study on the impact of hospital volume.	2011	Netherlands	Observational study	Low volume < 10 cystectomies per year, high volume ≥ 10 cystectomies per year.	Newly diagnosed MIBC patients (n = 13 033)	The risk of death < 30 days after radical cystectomy was significantly increased for T2/T3 patients in low‐volume hospitals when compared to high‐volume hospitals (HR = 1.17, 95% CI: 1.01‐1.35)	+
Mayer et al.	(30)	The volume‐mortality relation for radical cystectomy in England: retrospective analysis of hospital episode statistics.	2010	UK	Observational study	Annual cystectomy rate. Low > 2 and < 10; medium ≥ 10 and < 16; high ≥ 16.	Patients with a primary diagnosis of cancer undergoing an inpatient elective cystectomy. Number of patients not stated.	When compared to low‐volume centers, medium‐volume centers had significantly increased odds of both overall and in‐hospital mortality within 30 days post‐cystectomy. The magnitude of these odds varied depending on which variables were adjusted for in the model	+
Birkmeyer et al.	(32)	Hospital volume and late survival after cancer surgery.	2007	USA	Observational study	Hospital volume was measured for each of the 6 procedures in the study then collated into three groups: low, medium, and high.	2513 BC patients undergoing major resection (NMIBC and MIBC)	5‐year survival estimates for low‐volume vs. high‐volume centers were 35.4% and 39.0%	+
Udovicich et al.	(34)	Hospital volume and perioperative outcomes for radical cystectomy: a population study	2017	Australia	Observational study	Number of radical cystectomies per year. Low‐volume (<4), medium‐volume (4, 5, 6, 7, 8, 9, 10) and high volume (>10).	803 radical cystectomy patients	Low‐volume hospitals were associated with an increased risk of in‐house mortality (OR = 5.74, 95% CI: 1.06‐31.20)	+
Scarberry et al.	(35)	Improved surgical outcomes following radical cystectomy at high‐volume centers influence overall survival	2018	USA	Observational study	Academic vs. community cancer center status and radical cystectomy volume	39 274 radical cystectomy patients	Patients undergoing radical cystectomy at an academic center decreased risk of death (HR = 0.92, 95% CI:0.89‐0.95). A hospital volume of ≥ 10 cystectomies per year also reduced risk of death (HR = 0.91, 95% CI:0.87‐0.95)	+
Hounsome et al	(36)	Trends in operative caseload and mortality rates after radical cystectomy for bladder cancer in England for 1998‐2010	2015	UK	Observational study	Number of radical cystectomies	16 033 NMIBC and MIBC patients who underwent radical cystectomy, cystoprostatectomy, or cystourethrectomy.	Centralization of services has occurred over time along with a decrease in mortality (though this association was not quantified)	+
Liedberg et al.	(37)	Period‐specific mean annual hospital volume of radical cystectomy is associated with outcome and perioperative quality of care: a nationwide population‐based study	2019	Sweden	Observational study	Period‐specific mean annual hospital volume of cystectomies	5579 radical cystectomy patients	Patients treated at a hospital with a mean annual volume of ≥ 25 radical cystectomies (highest tertile) was associated with improved overall survival (HR = 0.83, 95% CI:0.71–0.98)	+
Afshar et al.	(38)	Centralization of radical cystectomies for bladder cancer in England, a decade on from the “Improving Outcomes Guidance”: the case for super centralization	2018	UK	Observational study	Number of cystectomies per center per year	15 292 radical cystectomy patients	Each single extra surgery per center reduced the odds of death at 30 days by 1.5% (odds ratio [OR] 0.985, 95% confidence interval [CI] 0.977–0.992) and 1% at 1 year (OR 0.990, 95% CI 0.988–0.993)	+
Williams et al.	(39)	Impact of Centralizing Care for Genitourinary Malignancies to High‐volume Providers: A Systematic Review	2019	N/A	Systematic review	Hospital volume/type as defined by each study	379 313 radical cystectomy patients (from various studies)	Most studies reported better survival outcomes and lower morbidity for high‐volume compared to low‐volume hospitals	+
Leow et al.	(40)	Impact of surgeon volume on the morbidity and costs of radical cystectomy in the USA: A contemporary population‐based analysis	2015	USA	Observational study	Annual number of radical cystectomies per surgeon	49 540 radical cystectomy patients	90‐day postoperative mortality rates decreased as surgeon volume increased (4.3% for very‐low‐volume surgeons and 2.4% for very‐high‐volume surgeons, *P* < .001)	+
McCabe et al.	(41)	Radical cystectomy: Defining the threshold for a surgeon to achieve optimum outcomes	2007	UK	Observational study	Number of radical cystectomies per year	6308 radical cystectomy patients	Inverse association between case volume and mortality (Pearson coefficient 20.968, *P* < .01)	+
de Vries	(42)	Outcome of treatment of bladder cancer: A comparison between low‐volume hospitals and an oncology center	2010	Amsterdam	Observational study	Annual number of radical cystectomies per hospital (Low < 5, medium 5‐10 and high volume > 10)	1185 radical cystectomy patients	30‐day post‐operative mortality was 1.8% in the high volume center and 3.5% in low volume centers though this was not significant	Null
Lieberman‐Cribbin et al.	(43)	Hospital Centralization Impacts High‐Risk Lung and Bladder Cancer Surgical Patients	2017	USA	Observational study	Number of radical cystectomies per year in quartiles (0–1.40, 1.41–2.00, 2.01–3.25 and > 3.25)	8160 radical cystectomy patients	In house mortality was 3.7% in the lowest quartile vs. 1.8% in the highest quartile (*P* < .001)	+

Note

95% CI – 95% confidence interval; BC, bladder cancer; HR, hazard ratio; MIBC, muscle‐invasive bladder cancer; RT, radiotherapy. “+” denotes positive association (ie higher hospital volume associated with increased survival or decreased risk of death), “null” denotes a null association.

Eleven of the 13 observational studies concluded that either a larger hospital volume (as defined by each study) was associated with increased survival, or a smaller hospital volume (as defined by each study) was associated with a decreased survival.^30^, ^31^, ^32^, ^43^, ^44^ The definition for a high volume center ranged from > 3.25 to 25‐66 cystectomies per year. The study by Bajaj et al^33^ utilized academic vs non‐academic center type as the exposure variable. They concluded that treatment with curative radiation therapy or concurrent chemoradiation therapy at an academic hospital was not associated with improved overall survival when compared to the same treatment at a non‐academic hospital. Moreover, the study by de Vries et al^42^ looked at 30‐day mortality post‐radical cystectomy between high and low volume centers (1.8% vs 3.5%, respectively) but deemed the difference non‐significant. The results from the studies within the review by Nuttal et al were mixed with some stating an association between hospital volume and survival while others did not.^29^ However, the authors state that “on average” patients undergoing a radical cystectomy at a high‐volume center have increased survival compared to those at a low‐volume center. The other review by Williams et al,^39^ which was published 15 years after the Nuttal review, concluded that most studies reported better survival outcomes in high volume centers.

### 3.3 Comorbidities and survival

709 studies were extracted from the search of which 19 were deemed suitable for inclusion (Table 3). These studies included 1 review^45^ and 18 cohort studies^46^, ^47^, ^48^, ^49^, ^50^, ^51^, ^52^, ^53^, ^54^, ^55^, ^56^, ^57^, ^58^, ^59^, ^60^, ^61^, ^62^, ^63^ (of which eight were from single‐center data)^63^). Thirteen of the 19 studies investigated the association between comorbidity and survival in patients undergoing radical cystectomy, one of which also looked at MIBC patients who had undergone external beam or interstitial radiotherapy.^53^ Three studies looked at this association in both NMIBC and MIBC patients; however, the study by Safarti et al^51^ did not stratify the analyses by NMIBC/MIBC and so the results could not be presented separately. Two studies looked at NMIBC patients only and the remaining study looked at patients treated with TURBT; the latter study did not describe more detailed stage information beyond NMIBC.^46^ None of the studies investigating comorbidities and survival included an SES indicator in their analyses.

**TABLE 3:** Studies identified relating to comorbidities and survival

Authors	Reference	Title	Year	Country	Study type	Patients	Comorbidity indicator	Summary of results	Association
Williams et al.	(45)	Systematic Review of Comorbidity and Competing‐risks Assessments for Bladder Cancer Patients	2018	USA	Review	N/A	ASA, ACE‐27, CCI, ECOG PS, KPS, and EI	Patients undergoing RC with high‐risk comorbidity and performance scores are up to seven times more likely to die from any cause compared to those with low scores. The studies in the review consistently demonstrate that patients with higher comorbidity have worse outcomes. The authors, therefore, conclude that the comorbidity risk assessment tool should be incorporated into pre‐operative treatment counseling	+
Pereira et al	(46)	The Perioperative Morbidity of Transurethral Resection of Bladder Tumor: Implications for Quality Improvement	2019	USA	Observational study	24 100 patients, aged 18‐89 who underwent TURBT	ASA score, diabetes, chronic obstructive pulmonary disease, congestive heart failure, hypertension requiring medical treatment, renal failure or dialysis, and bleeding disorder.	An ASA score of 3, or 4‐5 was associated with increased odds for 30‐day mortality when compared to an ASA score of 1‐2 (OR = 4.58, 95% CI: 2.57‐8.18 and OR = 12.29, 95% CI: 6.44‐23.46 respectively). Furthermore, having a dependent functional status, chronic heart failure and renal failure were also associated with perioperative mortality (OR = 3.21, 95% CI: 2.13‐4.84; OR = 2.09, 95% CI: 1.12‐3.90; 5.46, 95% CI: 3.28‐9.10 respectively)	+
Racioppi et al.	(47)	The challenges of Bacillus of Calmette‐Guerin (BCG) therapy for high‐risk non‐muscle‐invasive bladder cancer treatment in older patients	2018	Italy	Observational study	Newly diagnosed high‐grade NMIBC, aged > 80 (n = 200)	WHO PS, ASA, and CCI	No statistically significant difference in cancer‐free survival between the two groups (with varying comorbidities). The rate of overall complication was however higher in those with fewer comorbidities but they had BCG therapy more often as they were deemed more suitable for a more intense regime	Null
Froehner et al	(48)	Predicting 90‐day and long‐term mortality in octogenarians undergoing radical cystectomy	2018	Germany	Observational study	Patients with high‐risk NMIBC, MIBC or undifferentiated carcinoma of bladder who underwent RC (n = 1184)	ASA and CCI	Patients < 80 years old, CCI and ASA scores predicted 90‐day mortality however those aged > 80, only their age was an independent predictor	+
Johnson et al.	(49)	Perioperative and long‐term outcomes after radical cystectomy in hemodialysis patients	2018	USA	Observational study	Patients on hemodialysis who underwent RC (n = 985)	A history of hypertension, diabetes, coronary artery disease, congestive heart failure, chronic obstructive pulmonary disease, and cerebrovascular disease (CVD).	Among patients with end‐stage renal disease, age, diabetes, and CVD were associated with an increased hazard of all‐cause mortality. Age (HR = 1.02; 95% CI: 1.02‐1.03), diabetes (HR = 1.33 95% CI: 1.10–1.61), and CVD (1.48; 95% CI: 1.01– 2.18). Active smoking was the sole risk factor for cancer‐specific mortality	+
Dell'Oglio et al.	(50)	Short‐form Charlson Comorbidity Index for assessment of perioperative mortality after radical cystectomy	2017	Canada	Observational study	Non‐metastatic BC patients treated with RC (n = 10 522)	Deyo adaptation of CCI (DaCCI)	The authors created a short‐form of the Deyo adaptation of the CCI using just 3 of the original 17 comorbid condition groupings (congestive heart failure, CVD, and chronic pulmonary disease). The aim was to make this more specific to BC patients undergoing RC. The accuracy for 90‐day mortality post RC was slightly higher in the short form version	+
Sarfati et al.	(51)	Identifying important comorbidity among cancer populations using administrative data: Prevalence and impact on survival	2016	New Zealand	Observational study	Patients newly diagnosed with nine different types of cancer (BC patients, n = 647)	50 comorbid conditions derived from CCI, ACE‐27, Elixhauser, and seven other validated comorbidity indices used within cancer.	Patients with BC tended to be older and had an increased rate of comorbidities associated with smoking. Almost all of the comorbidities were associated with an increased hazard for all‐cause mortality in urological cancers (renal and bladder)	+
Li et al.	(63)	Chronic kidney disease as an important risk factor for tumor recurrences, progression, and overall survival in primary non‐muscle‐invasive bladder cancer	2016	Taiwan	Single‐center, observational study	Newly diagnosed NMIBC patients (n = 158)	Chronic kidney disease (CKD)	CKD in NMIBC was associated with higher tumor recurrence, progression rates, and hazard of overall survival than patients without CKD. CKD was not associated with increased odds of BC‐specific survival. The authors suggest the kidneys and ureters should be surveyed every 3‐6 months and a second TURBT should be considered in these patients to monitor progression	+
Dybowski et al.	(52)	Impact of stage and comorbidities on five‐year survival after radical cystectomy in Poland: Single center experience	2015	Poland	Single‐center, observational study	MIBC patients who have undergone RC (n = 63)	Six individual comorbid conditions and “significant comorbidity” were investigated	Comorbidities were not an independent predictor of 5‐year overall survival. However, a combination of stage, diabetes status, and postoperative course was	Null
Goossens‐Laan et al.	(53)	Effects of age and comorbidity on treatment and survival of patients with muscle‐invasive bladder cancer	2014	Netherlands	Observational study	MIBC patients (n = 2445)	Modified Charlson score	Comorbidity was an independent predictor of overall survival (two or more comorbid conditions: HR = 1.4, 95% CI: 1.1‐1.5). Diabetes (HR: 1.5, 95% CI: 1.3–1.8), cardiovascular disease (HR: 1.3, 95% CI: 1.2–1.5), hypertension (HR: 1.1, 95% CI: 1.0–1.3) and pulmonary disease (HR: 1.5, 95% CI: 1.3–1.7) were each found to be independently associated with overall survival	+
Mayr et al.	(54)	Comorbidity and performance indices as predictors of cancer‐independent mortality but not of cancer‐specific mortality after radical cystectomy for urothelial carcinoma of the bladder.	2012	Germany	Observational study	Patients who have undergone RC (n = 555)	ASA, ECOG, ACE‐27, CCI, ACCI	ASA, ECOG, ACE27, CCI, and ACCI were all positively associated with in increased risk in cancer‐independent mortality after radical cystectomy. ASA (3, 4), ECOG (2, 3), ACE27 (3), and ACCI (>5) were all associated with an increased risk of cancer‐specific mortality after radical cystectomy	+
Mayr et al.	(55)	Predictive capacity of four comorbidity indices estimating perioperative mortality after radical cystectomy for urothelial carcinoma of the bladder	2012	Germany	Observational study	Patients who have undergone RC (n = 555)	ACE‐27, CCI, ECOG, and ASA	The four comorbidity indices were assessed to see which ones correlated correlate with perioperative mortality 90 days after RC. All four were independent predictors of 90‐day mortality and increased the predictive capacity of the basic model using clinical variables. However, ASA and ACE‐27 increased the predictive capacity the most (by 28.3% and 29.8% respectively)	+
Lund et al.	(56)	Impact of comorbidity on survival of invasive bladder cancer patients, 1996‐2007: A danish population‐based cohort study	2010	Denmark	Observational study	MIBC who have undergone RC (n = 3997)	CCI	Across all the study periods, an increase in comorbidity resulted in an increase in mortality rate for one, three and five‐year survival	+
Ha and Chang	(57)	Significance of age and comorbidity as prognostic indicators for patients with bladder cancer	2010	Korea	Single‐center observational study	Newly diagnosed BC patients (n = 528)	ACE‐27	Older patients had more comorbidities. Moderate to severe comorbidity status was predictive of lower overall and cancer‐specific survival when considering the whole cohort (HR = 1.87, 95% CI: 1.40–2.51; HR = 1.70, 95%CI: 1.15‐2.53) respectively). When stratified by invasiveness, this association remained among MIBC patients but only for overall survival in NMIBC patients	+
Koppie et al.	(58)	Age‐adjusted Charlson comorbidity score is associated with treatment decisions and clinical outcomes for patients undergoing radical cystectomy for bladder cancer.	2008	USA	Single‐center observational study	Patients who have undergone RC (n = 1121)	ACCI	Higher ACCI was associated with lower overall (*P* < .005) but not recurrence‐free survival after RC (*P* = .17)	+
Megwalu et al.	(59)	Prognostic impact of comorbidity in patients with bladder cancer.	2008	USA	Single‐center observational study	Newly diagnosed BC patients (n = 675)	ACE‐27	ACE‐27 independently predicted overall survival in all BC patients. Those with moderate and severe levels of comorbidity in the NMIBC group were associated with an increased risk in overall survival, while only severe comorbidity was a predictor in those treated with cystectomy	+
Zhu et al	(60)	Comorbidity relationship to outcome of radical cystectomy in Chinese: a single‐institution study with the ACE‐27 comorbidity index.	2012	China	Single‐center observational study	Patients who have undergone RC (n = 246)	ACE‐27	Patients with moderate (*P* = .002) and severe (*P* < .001) comorbidities were associated with experiencing a decreased overall survival	+
Miller et al.	(61)	The impact of the co‐morbid disease on cancer control and survival following radical cystectomy.	2003	USA	Single‐center observational study	Patients who have undergone RC with curative intent (cT2 or less) (n = 106)	CCI	CCI was independently associated with a reduced odds of the disease remaining confined to the bladder (OR = 0.66, 95% CI: 0.45‐0.97) and increased hazard of cancer‐specific death (HR = 1.26, 95% CI:1.00‐1.58). CCI was not however associated with overall death	+
Boorjian et al.	(62)	Comparative Performance of Comorbidity Indices for Estimating Perioperative and 5‐Year All Cause Mortality Following Radical Cystectomy for Bladder Cancer	2013	USA	Single‐center observational study	Patients who had undergone RC (n = 891)	ASA, CCI, EI and ECOG	ASA (HR = 3.17, *P* = .001), EI (HR = 1.48, *P* = .002) and ECOG (HR = 2.40, *P* < .001) were all associated with risk of perioperative mortality (within 90‐days). All four indices were independent predictors of 5‐year all‐cause mortality: CCI (HR = 1.23, *P* < .0001), EI (HR = 1.28, *P* < .0001), ASA (HR = 1.44, *P* = .007) and ECOG (HR = 1.97, *P* < .0001)	+

Note

95% CI, 95% confidence interval; ACCI, age‐adjusted CCI; ACE‐27, Adult Comorbidity Evaluation‐27; ASA, American Society of Anesthesiologists; BC, bladder cancer; CCI, Charlson Comorbidity Index; CVD, cardiovascular disease; ECOG PS, Eastern Cooperative Oncology Group Performance Status; EI, Elixhauser index; HR, hazard ratio; MIBC, muscle‐invasive bladder cancer; NMIBC, non‐muscle‐invasive bladder cancer; OR–odds ratio; RC, radical cystectomy; SES, socioeconomic status; WHO PS, World Health Organization Performance Status. “+” denotes positive association (ie increasing comorbidity index/number associated with increased risk of death), “null” denotes a null association.

For all MIBC patients and those undergoing curative treatment (radical cystectomy or external beam radiotherapy), 12 of the studies concluded an association between increasing comorbidity (as defined by each study) and an increased risk of overall death.^48^, ^49^, ^50^, ^62^ Three studies stated the same direction of association for cancer‐specific death.^54^, ^57^, ^61^

The ways in which the studies measured comorbidity for the radical cystectomy patients varied as can be seen in Table 3. Some examples of measurements used include CCI, age‐adjusted CCI (ACCI), Adult Comorbidity Evaluation‐27 (ACE‐27), American Society of Anesthesiologists (ASA), Eastern Cooperative Oncology Group Performance Status (ECOG PS), and Elixhauser index (EI). A common theme among several of the studies was the comparison of the predictive value of these comorbidity indices on survival.^48^, ^54^, ^55^, ^62^ The most recent of the two studies by Mayr et al investigated ASA, ECOG, ACE‐27, CCI, and ACCI.^54^ Increasing values of all five comorbidity indicators were associated with cancer independent mortality post‐cystectomy. Furthermore, ASA (score of 3‐4), ECOG,^2^, ^3^ ACE27,^3^ and ACCI (>5) were all associated with an increased risk of cancer‐specific mortality after radical cystectomy. Similarly, Boorjian et al compared the predictive capabilities of CCI, ASA, ECOG, and EI indices on perioperative and 5‐year all‐cause mortality in radical cystectomy patients.^62^ They concluded that all four indices were independent predictors of 5‐year mortality, but only ASA, ECOG, and EI were predictors of perioperative mortality (within 90‐days post‐cystectomy).

Three of the studies which included NMIBC patients described a positive association between comorbidity and an increased risk of overall death.^57^, ^59^, ^63^ None of the studies investigating NMIBC patients concluded the same association when considering cancer‐specific death. Although Sarfati et al did not stratify their analyses by NMIBC and MIBC patients, they noted a positive association between almost all the comorbidities investigated with overall mortality in urological cancers (renal and bladder).^51^

Two studies specifically investigated chronic kidney disease (CKD) as a major contributing factor to the link between comorbidity status and survival in bladder cancer patients.^49^, ^63^ The study by Li et al encompassed NMIBC patients who underwent TURBT, both with and without CKD.^63^ The authors concluded that bladder cancer patients with CKD had increased odds of all‐cause death when compared to patients without CKD.

### 3.4 SES and treatment delay

Two studies were deemed suitable for inclusion when investigating the relationship between SES and treatment delay^1^, ^5^ (Table 4). The study by Jacobs et al was a more general review looking into the disparities in survival in bladder cancer patients. The authors delved into factors such as SES and access to care stating that patients from a lower SES group may have their treatments delayed due to a variety of factors such as weak social support, lack of transportation, and cultural behaviors.^1^ There was, however, no quantification of this association available. Meanwhile, the study by Begum et al^5^ concluded that there were no differences in delay times among socio‐economic groups for the delay categories of symptom onset to first referral, referral to first attendance at hospital, first hospital attendance to first treatment (TURBT), and the total delay from onset of symptoms to first treatment.

**TABLE 4:** Studies identified relating to socioeconomic status and treatment delay

Authors	Reference	Title	Year	Country	Study type	SES indicator	Summary of results
Jacobs et al.	(1)	Disparities in bladder cancer	2012	USA	Review	Income, occupation, education, extent of health insurance	Weak social support, lack of transportation, and behavioral differences in lower SES groups may contribute to a delay in receiving treatment. Countries that require medical insurance e.g. America, often see patients with a lower SES unable to reach out for medical care
Begum et al.	(5)	Socio‐economic deprivation and survival in bladder cancer	2004	UK	Observational Study	Townsend score	No significant difference in delay times among socio‐economic groups for all delay categories (*P* = .16)

Note

SES, socioeconomic status.

### 3.5 Quality of the included studies

Overall, the majority of the observational studies assessed using the ROBINS‐I tool were deemed to have a low risk of bias (Tables S1‐S3, Appendix). A few had a moderate risk of bias; however, these tended to be the studies that had not sufficiently adjusted for all possible confounding variables or stated crude survival proportions only. Using the AMSTAR criteria, there was deemed to be a moderate level of confidence in the results from the four systematic reviews (Figure S1, Appendix). There was, however, a paucity in pre‐defined study protocols detailing the search, definition of inclusion and exclusion criteria, and all failed to assess the risk of bias of the studies within each relative review. The two narrative reviews were both deemed of good quality according to the SANRA criteria (Figure S2, Appendix).

## 4 DISCUSSION

This review collated information from existing literature about the potential mediators (hospital type, comorbidities, and treatment delay) for the association between SES and survival in bladder cancer patients by specifically evaluating how each of them is associated with SES and/or bladder cancer survival. Overall, the review suggested associations between each of the three potential mediators with survival. However, there was a paucity of information relating to the association between SES and the mediators investigated.

One notable finding was the heterogeneity in which SES was assessed. However, there is no perfect way to assess SES levels and the method used by a certain study may be limited by the variables available in a particular dataset. An example of another possible proxy for SES is educational level; however, this was not utilized by any of the studies in this review. Despite the use of several types of SES indicators, the results from the current review suggest that a lower SES is associated with reduced survival. The only study which did not reach the same conclusion was by Eberle et al which used a two‐step method to determine SES based on an existing discrimination index for Bremen.^19^

### 4.1 Hospital type

All of the 15 studies which investigated the association between hospital type and overall survival defined the hospital type in terms of hospital volume, while two additionally assessed academic vs non‐academic centers. No studies were found to investigate SES and hospital type for bladder cancer specifically; however, a study by Chang et al investigated this research question for breast cancer patients.^64^ They concluded that patients with a lower SES were more likely to be treated at a low‐volume hospital and be operated on by a low‐volume surgeon. Therefore, to establish whether a similar association exists in bladder cancer patients, future studies are needed that also adjust for the interaction with other factors such as distance to treatment facilities.^65^

### 4.2 Comorbidity

Although no studies looking at SES and comorbidity were identified, outside of the bladder cancer setting evidence does exist for an association between these two variables. For example, a more generalized systematic review and meta‐analysis^66^ identified 24 cross‐sectional studies and found SES to be assessed by many measures including education, deprivation, income, occupational social class, social class (not defined), literacy score, employment status, and self‐reported poverty. Low education was associated with 64% increased odds of multimorbidity (OR: 1.64, 95% CI: 1.41‐1.91); deprivation was also linked to risk of multimorbidity, although the evidence for income was mixed. The authors also stated that there was heterogeneity in the assessment of multimorbidity across the studies.

We also observed heterogeneity in the methods for assessing comorbidity among the studies. Even though many methods exist, results from this review would suggest that most measures of comorbidity have predictive capabilities regarding survival in bladder cancer patients who have undergone radical cystectomy.

There was also heterogeneity in the patients included within the studies. Most of the studies investigated patients who had undergone radical cystectomy, but this included both NMIBC and MIBC patients. Other studies included NMIBC patients only or patients who had undergone TURBT (with no information about their staging). This may have had an effect on the type of comorbidity measures which were utilized by the studies.

### 4.3 Treatment delay

The paucity of studies investigating SES and treatment delay, together with the literature on SES, diagnostic delay, and variation in emergency presentation^67^ suggests a complex mediation of SES and survival in bladder cancer. Thus, more advanced disease at diagnosis in patients with lower SES affects further treatment possibilities.^68^

### 4.4 Strengths and limitations

Several of the studies, in particular those investigating the association between comorbidities and survival, were single‐center studies.^52^, ^57^, ^58^, ^59^, ^60^, ^61^, ^62^, ^63^ Possible reasons for this include the lack of a universal comorbidity score and that different scores perform better from one disease or treatment or surgery to another. These studies may not have the same degree of external validity seen in nationwide or larger studies. Nonetheless, single‐center studies are still crucial in healthcare research as they can capture data on a more granular level when compared to larger cohort studies. This review additionally benefitted from the inclusion of many large cohort studies. These studies are inclusive of heterogeneous populations, which is especially important when studying factors such as SES.

A large proportion of the studies, particularly when looking at comorbidities, included patients undergoing radical cystectomy. Therefore, a limitation to the current review is the low number of studies that included NMIBC patients, or those undergoing alternate treatments. Some studies did, however, include patients undergoing radiotherapy. It is important to note, however, that this may also be a limitation since patients who receive radiotherapy often do so as a result of being too frail for surgery and hence may skew the results for survival. Furthermore, none of the studies within this review used a formal mediation analysis. It is also possible that some literature may have been missed if studies included the research question of interest as a secondary or tertiary research question and were subsequently not picked up during title and abstract screening.

Meta‐analyses were not deemed suitable in this review due to the heterogeneity of the studies identified.

## 5 CONCLUSIONS

The studies identified in this review imply associations between the possible mediators of the association between SES and survival in bladder cancer patients (hospital type, comorbidities, and treatment delay). While a low SES was found to be associated with decreased survival in numerous studies (despite heterogeneous methods used to assess SES), this review has highlighted a paucity of studies investigating mediators for this association. Further studies investigating the relationship between the proposed mediators and SES using logistic regression models are warranted for a deeper understanding of the relationship between SES and survival. With such an understanding, modifiable mediators, eg, treatment delay, may be identified and further motivate the standardization of clinical care across SES groups.

## CONFLICT OF INTEREST

RT Bryan has contributed to advisory boards for Olympus Medical Systems & Janssen, and undertakes research funded by UroGen Pharma and QED Therapeutics. We can confirm all other authors have no conflicts of interest to declare.

## Supporting information

Supplementary Material

## References

[1] Jacobs BL , Montgomery JS , Zhang Y , Skolarus TA , Weizer AZ , Hollenbeck BK . Disparities in bladder cancer. Urol Oncol. 2012;30:81–8. 22127016 10.1016/j.urolonc.2011.08.011

[2] Bryere J , Dejardin O , Launay L , Colonna M , Grosclaude P , Launoy G . Socioeconomic status and site‐specific cancer incidence, a Bayesian approach in a French Cancer Registries Network study. Eur J Cancer Prev. 2016;27(4):1. 10.1097/CEJ.0000000000000326 27879493

[3] Bryere J , Dejardin O , Bouvier V , Colonna M , Guizard A‐V , Troussard X , et al. Socioeconomic environment and cancer incidence: a French population‐based study in Normandy. BMC Cancer. 2014;14:1–10. 24524213 10.1186/1471-2407-14-87 PMC3930294

[4] Woods LM , Rachet B , Coleman MP . Origins of socio‐economic inequalities in cancer survival: a review. Ann Oncol. 2006;17:5–19. 16143594 10.1093/annonc/mdj007

[5] Begum G , Dunn JA , Bryan RT , Bathers S , Wallace DMA . Socio‐economic deprivation and survival in bladder cancer. BJU Int. 2004;1(94):539–43. 10.1111/j.1464-410X.2004.04997.x 15329108

[6] Mackillop W , Zhang‐Salomons J , Groome P , Paszat L , Holowaty E . Socioeconomic status and cancer survival in Ontario. J Clin Oncol. 1997;15(4):1680–9. 9193369 10.1200/JCO.1997.15.4.1680

[7] Babjuk M , Böhle A , Burger M , Compérat E , Kaasinen E , Palou J . (2019). EAU guidelines: Non‐muscle invasive bladder cancer [Internet]. Available from: https://uroweb.org/guideline/non‐muscle‐invasive‐bladder‐cancer/#1

[8] Witjes JA , Bruins M , Compérat E , Cowan NC , Gakis G , Hernández V . (2019).EAU guidelines: Muscle‐invasive and metastatic bladder cancer [Internet]. Available from: https://uroweb.org/guideline/bladder‐cancer‐muscle‐invasive‐and‐metastatic/#7

[9] Quaglia A , Lillini R , Mamo C , Ivaldi E , Vercelli M . Socio‐economic inequalities: a review of methodological issues and the relationships with cancer survival. Crit Rev Oncol Hematol. 2013;85:266–77. 22999326 10.1016/j.critrevonc.2012.08.007

[10] Russell B , Hemelrijck MV , Gårdmark T , Holmberg L , Kumar P , Bellavia A , et al. A mediation analysis to explain socio‐economic differences in bladder cancer survival. Cancer Med. 2020:1–11. 10.1002/cam4.3418 PMC7571835 32851811

[11] Shanmugalingam T , Crawley D , Bosco C , Melvin J , Rohrmann S , Chowdhury S , et al. Obesity and cancer: the role of vitamin D. BMC Cancer. 2014;14(712). 10.1186/1471-2407-14-712 PMC4182855 25255691

[12] Russell B , Liedberg F , Khan MS , Nair R , Thurairaja R , Malde S , et al. A systematic review and meta‐analysis of delay to radical cystectomy and the effect on survival in bladder cancer patients. Eur Urol Oncol. 2020;3(2):239–49. 31668714 10.1016/j.euo.2019.09.008

[13] Liberati A , Altman DG , Tetzlaff J , Mulrow C , Gøtzsche PC , Ioannidis JPA , et al. The PRISMA statement for reporting systematic reviews and meta‐analyses of studies that evaluate health care interventions: explanation and elaboration. PLoS Med. 2009;6(7):e1000100. 19621070 10.1371/journal.pmed.1000100 PMC2707010

[14] Sterne JA , Hernán MA , Reeves BC , Savović J , Berkman ND , Viswanathan M , et al. ROBINS‐I: a tool for assessing risk of bias in non‐randomised studies of interventions. BMJ. 2016;12(355):i4919. 10.1136/bmj.i4919 PMC5062054 27733354

[15] Shea BJ , Reeves BC , Wells G , Thuku M , Hamel C , Moran J , et al. AMSTAR 2: a critical appraisal tool for systematic reviews that include randomised or non‐randomised studies of healthcare interventions, or both. BMJ. 2017;358:j4008. 28935701 10.1136/bmj.j4008 PMC5833365

[16] Baethge C , Goldbeck‐Wood S , Mertens S . SANRA—a scale for the quality assessment of narrative review articles. Res Integr Peer Rev. 2019;4:5. 30962953 10.1186/s41073-019-0064-8 PMC6434870

[17] Shackley DC , Clarke NW . Impact of socioeconomic status on bladder cancer outcome. Curr Opin Urol. 2005;15(5):328–31. 16093857 10.1097/01.mou.0000174965.26439.d1

[18] Lara J , Brunson A , Keegan TH , Malogolowkin M , Pan CX , Yap S , et al. Determinants of survival in adolescents and young adults with urothelial bladder cancer: results from the California Cancer Registry. J Urol. 2016;196(5):1378–82. 27208515 10.1016/j.juro.2016.05.082 PMC5319865

[19] Eberle A , Luttmann S , Foraita R , Pohlabeln H . Socioeconomic inequalities in cancer incidence and mortality—A spatial analysis in Bremen, Germany. J Public Heal. 2010;18(3):227–35.

[20] Sloggett A , Young H , Grundy E . The association of cancer survival with four socioeconomic indicators: a longitudinal study of the older population of England and Wales 1981–2000. BMC Cancer. 2007;7:20. 17254357 10.1186/1471-2407-7-20 PMC1797185

[21] Klapheke A , Yap SA , Pan K , Cress RD . Sociodemographic disparities in chemotherapy treatment and impact on survival among patients with metastatic bladder cancer. Urol Oncol Semin Orig Investig. 2018;36(6):308.e19–308.e25. 10.1016/j.urolonc.2018.03.008 PMC5960436 29628318

[22] Chien LH , Tseng TJ , Tsai FY , Wang JH , Hsiung CA , Liu TW , et al. Patterns of age‐specific socioeconomic inequalities in net survival for common cancers in Taiwan, a country with universal health coverage. Cancer Epidemiol. 2018;53:42–8. 29396159 10.1016/j.canep.2018.01.006

[23] Belot A , Remontet L , Rachet B , et al. Describing the association between socioeconomic inequalities and cancer survival: methodological guidelines and illustration with population‐based data. Clin Epidemiol. 2018;10:561–73. 29844706 10.2147/CLEP.S150848 PMC5961638

[24] Syriopoulou E , Bower H , Andersson TML , Lambert PC , Rutherford MJ . Estimating the impact of a cancer diagnosis on life expectancy by socio‐economic group for a range of cancer types in England. Br J Cancer. 2017;117:1419–26. 28898233 10.1038/bjc.2017.300 PMC5672926

[25] Sundquist J , Li X , Sundquist K . Neighborhood deprivation and mortality in individuals with cancer: a multilevel analysis from Sweden. Eur J Cancer Prev. 2012;21(4):387–94. 22495253 10.1097/CEJ.0b013e32834dbc2e

[26] Shack LG , Rachet B , Brewster DH , Coleman MP . Socioeconomic inequalities in cancer survival in Scotland 1986–2000. Br J Cancer. 2007;97(7):999–1004. 17876331 10.1038/sj.bjc.6603980 PMC2360415

[27] Moran A , Sowerbutts A‐M , Collins S , Clarke N , Cowan R . Bladder cancer: worse survival in women from deprived areas. Br J Cancer. 2004;90:2142–4. 15150549 10.1038/sj.bjc.6601847 PMC2409491

[28] Coleman MP , Rachet B , Woods LM , Mitry E , Riga M , Cooper N , et al. Trends and socioeconomic inequalities in cancer survival in England and Wales up to 2001. Br J Cancer. 2004;90:1367–73. 15054456 10.1038/sj.bjc.6601696 PMC2409687

[29] Nuttall M , van der Meulen J , Phillips N , Sharpin C , Gillatt D , McIntosh G , et al. A systematic review and critique of the literature relating hospital or surgeon volume to health outcomes for 3 urological cancer procedures. J Urol. 2004;172(6 Pt 1):2145–52. 15538220 10.1097/01.ju.0000140257.05714.45

[30] Mayer EK , Bottle A , Darzi AW , Athanasiou T , Vale JA . The volume‐mortality relation for radical cystectomy in England: retrospective analysis of hospital episode statistics. BMJ. 2010;340:c1128. 20305302 10.1136/bmj.c1128 PMC2842924

[31] Goossens‐Laan CA , Visser O , Hulshof MCCM , Wouters MW , Bosch JLHR , Coebergh JWW , et al. Survival after treatment for carcinoma invading bladder muscle: a Dutch population‐based study on the impact of hospital volume. BJU Int. 2011;110(2):226–32. 22044615 10.1111/j.1464-410X.2011.10694.x

[32] Birkmeyer JD , Sun Y , Wong SL , Stukel TA . Hospital volume and late survival after cancer surgery. Ann Surg. 2007;245(5):777–83. 17457171 10.1097/01.sla.0000252402.33814.dd PMC1877074

[33] Bajaj A , Martin B , Bhasin R , Hentz C , Block AM , Harkenrider MM , et al. The impact of academic facility type and case volume on survival in patients undergoing curative radiation therapy for muscle‐invasive bladder cancer. Int J Radiat Oncol Biol Phys. 2018;100(4):851–57. 29485062 10.1016/j.ijrobp.2017.11.040

[34] Udovicich C , Perera M , Huq M , Wong LM , Lenaghan D . Hospital volume and perioperative outcomes for radical cystectomy: a population study. BJU Int. 2017;119:26–32. 28544301 10.1111/bju.13827

[35] Scarberry K , Berger NG , Scarberry KB , Agrawal S , Francis JJ , Yih JM , et al. Improved surgical outcomes following radical cystectomy at high‐volume centers influence overall survival. Urol Oncol Semin Orig Investig. 2018;36(6):308.e11–308.e17. 10.1016/j.urolonc.2018.03.007 29628316

[36] Hounsome LS , Verne J , McGrath JS , Gillatt DA . Trends in operative caseload and mortality rates after radical cystectomy for bladder cancer in England for 1998–2010. Eur Urol. 2015;67(6):1056–62. 25530608 10.1016/j.eururo.2014.12.002

[37] Liedberg F , Hagberg O , Aljabery F , Gardmark T , Hosseini A , Jahnson S , et al. Period‐specific mean annual hospital volume of radical cystectomy is associated with outcome and perioperative quality of care: a nationwide population‐based study. BJU Int. 2019;124(3):449–56. 30950568 10.1111/bju.14767

[38] Afshar M , Goodfellow H , Jackson‐Spence F , Evison F , Parkin J , Bryan RT , et al. Centralisation of radical cystectomies for bladder cancer in England, a decade on from the “Improving Outcomes Guidance”: the case for super centralisation. BJU Int. 2018;1(121):217–24. 10.1111/bju.13929 28594471

[39] Williams SB , Ray‐Zack MD , Hudgins HK , Oldenburg J , Trinh QD , Nguyen PL , et al. Impact of centralizing care for genitourinary malignancies to high‐volume providers: a systematic review. Eur Urol Oncol. 2019;2(3):265–73. 31200840 10.1016/j.euo.2018.10.006 PMC10007401

[40] Leow JJ , Reese S , Trinh QD , Bellmunt J , Chung BI , Kibel AS , et al. Impact of surgeon volume on the morbidity and costs of radical cystectomy in the USA: a contemporary population‐based analysis. BJU Int. 2015;115(5):713–21. 24674655 10.1111/bju.12749

[41] McCabe JE , Jibawi A , Javle PM . Radical cystectomy: defining the threshold for a surgeon to achieve optimum outcomes. Postgrad Med J. 2007;83(982):556–60. 17675551 10.1136/pgmj.2007.058214 PMC2600107

[42] de Vries RR , Visser O , Nieuwenhuijzen JA , Horenblas S . Outcome of treatment of bladder cancer: a comparison between low‐volume hospitals and an oncology centre. World J Urol. 2010;28(4):431–37. 20130885 10.1007/s00345-010-0512-z

[43] Lieberman‐Cribbin W , Galsky M , Casey M , Liu B , Oh W , Flores R , et al. Hospital centralization impacts high‐risk lung and bladder cancer surgical patients. Cancer Invest. 2017;35(10):652–61. 29243986 10.1080/07357907.2017.1406495

[44] Hemal AK , Kolla SB , Wadhwa P , Dogra PN , Gupta NP . Laparoscopic radical cystectomy and extracorporeal urinary diversion: a single center experience of 48 cases with three years of follow‐up. Urology. 2008;71(1):41–6. 18242362 10.1016/j.urology.2007.08.056

[45] Williams SB , Kamat AM , Chamie K , Froehner M , Wirth MP , Wiklund PN , et al. Systematic review of comorbidity and competing‐risks assessments for bladder cancer patients. Eur Urol Oncol. 2018;1(2):91–100. 30345422 10.1016/j.euo.2018.03.005 PMC6190914

[46] Pereira JF , Pareek G , Mueller‐Leonhard C , Zhang Z , Amin A , Mega A , et al. The perioperative morbidity of transurethral resection of bladder tumor: implications for quality improvement. Urology. 2019;1(125):131–7. 10.1016/j.urology.2018.10.027 30366045

[47] Racioppi M , Di Gianfrancesco L , Ragonese M , Palermo G , Sacco E . The challenges of Bacillus of Calmette‐Guerin (BCG) therapy for high risk non muscle invasive bladder cancer treatment in older patients. J Geriatr Oncol. 2018;9(5):507–12. 29673806 10.1016/j.jgo.2018.03.020

[48] Froehner M , Koch R , Hubler M , Heberling U , Novotny V , Zastrow S , et al. Predicting 90‐day and long‐term mortality in octogenarians undergoing radical cystectomy. BMC Urol. 2018;18(1):91. 30348141 10.1186/s12894-018-0402-z PMC6198515

[49] Johnson SC , Smith ZL , Golan S , Rodriguez JF , Pearce SM , Smith ND . Perioperative and long‐term outcomes after radical cystectomy in hemodialysis patients. Urol Oncol Semin Orig Investig. 2018;36(5):237. 10.1016/j.urolonc.2017.12.024 29395954

[50] Dell’Oglio P , Tian Z , Leyh‐Bannurah S‐R , Trudeau V , Larcher A , Moschini M , et al. Short‐form charlson comorbidity index for assessment of perioperative mortality after radical cystectomy. J Natl Compr Cancer Netw. 2017;15(3):327–33. 10.6004/jnccn.2017.0032 28275033

[51] Sarfati D , Gurney J , Lim B , Bagheri N , Simpson A , Koea J . Identifying important comorbidity among cancer populations using administrative data: prevalence and impact on survival. Asia Pac J Clin Oncol. 2016;12:e47‐e56. 24354451 10.1111/ajco.12130

[52] Dybowski B , Ossolinski K , Ossolinska A , Peller M , Bres‐Niewada E , Radziszewski P . Impact of stage and comorbidities on five‐year survival after radical cystectomy in Poland: single centre experience. Cent Eur J Urol. 2015;68(3):278‐83. 10.5173/ceju.2015.620 PMC4643711 26568866

[53] Goossens‐Laan CA , Leliveld AM , Verhoeven RHA , Kil PJM , de Bock GH , Hulshof MCCM , et al. Effects of age and comorbidity on treatment and survival of patients with muscle‐invasive bladder cancer. Int J Cancer. 2014;135(4):905–12. 24420527 10.1002/ijc.28716

[54] Mayr R , May M , Martini T , Lodde M , Comploj E , Pycha A , et al. Comorbidity and performance indices as predictors of cancer‐independent mortality but not of cancer‐specific mortality after radical cystectomy for urothelial carcinoma of the bladder. Eur Urol. 2012;62(4):662–70. 22534059 10.1016/j.eururo.2012.03.057

[55] Mayr R , May M , Martini T , Lodde M , Pycha A , Comploj E , et al. Predictive capacity of four comorbidity indices estimating perioperative mortality after radical cystectomy for urothelial carcinoma of the bladder. BJU Int. 2012;110(6B):E222–7. 22314129 10.1111/j.1464-410X.2012.10938.x

[56] Lund L , Jacobsen J , Clark P , Borre M , Nørgaard M . Impact of comorbidity on survival of invasive bladder cancer patients, 1996–2007: a Danish population‐based cohort study. Urology. 2010;75(2):393–8. 10.1016/j.urology.2009.07.1320 19914698

[57] Ha MS , Chang IH , Chang H . Significance of age and comorbidity as prognostic indicators for patients with bladder cancer. Nat Publ Gr. 2010;12:766–74. 10.1038/aja.2010.29 PMC3739310 20676116

[58] Koppie TM , Serio AM , Vickers AJ , Vora K , Dalbagni G , Donat SM , et al. Age‐adjusted Charlson comorbidity score is associated with treatment decisions and clinical outcomes for patients undergoing radical cystectomy for bladder cancer. Cancer. 2008;112(11):2384–92. 18404699 10.1002/cncr.23462

[59] Megwalu II , Vlahiotis A , Radwan M , Piccirillo JF , Kibel AS . Prognostic impact of comorbidity in patients with bladder cancer. Eur Urol. 2008;53(3):581–9. 17997024 10.1016/j.eururo.2007.10.069 PMC2262100

[60] Zhu X , Zhong ZH , Zhang XZ , Zhang L , Zhao XK , Lv C , et al. Comorbidity relationship to outcome of radical cystectomy in Chinese: a single institution study with the ACE‐27 comorbidity index. Asian Pac J Cancer Prev. 2012;13:827–31. 22631656 10.7314/apjcp.2012.13.3.827

[61] Miller DC , Taub DA , Dunn RL , Montie JE , Wei JT . The impact of co‐morbid disease on cancer control and survival following radical cystectomy. J Urol. 2003;169:105–9. 12478114 10.1016/S0022-5347(05)64046-3

[62] Boorjian SA , Kim SP , Tollefson MK , Carrasco A , Cheville JC , Thompson RH , et al. Comparative performance of comorbidity indices for estimating perioperative and 5‐year all cause mortality following radical cystectomy for bladder cancer. J Urol. 2013;190(1):55–60. 23313198 10.1016/j.juro.2013.01.010

[63] Li CE , Chien CS , Chuang YC , Chang YI , Tang HP , Kang CH , Chronic kidney disease as an important risk factor for tumor recurrences, progression and overall survival in primary non‐muscle‐invasive bladder cancer. Int Urol Nephrol. 2016;48(6):993–9. 26995008 10.1007/s11255-016-1264-5

[64] Chang C‐M , Yin W‐Y , Wei C‐K , et al.The association of socioeconomic status and access to low‐volume service providers in Breast Cancer. 2013. 10.1371/journal.pone.0081801 PMC3846901 24312589

[65] Ryan S , Serrell EC , Karabon P , Mills G , Hansen M , Hayn M , et al. The association between mortality and distance to treatment facility in patients with muscle invasive bladder cancer. J Urol. 2018;199(2):424–9. 29030318 10.1016/j.juro.2017.10.011

[66] Pathirana TI , Jackson CA . Socioeconomic status and multimorbidity: a systematic review and meta‐analysis. Aust N Z J Public Health. 2018;42(2):186–94. 29442409 10.1111/1753-6405.12762

[67] Abel GA , Shelton J , Johnson S , Elliss‐Brookes L , Lyratzopoulos G . Cancer‐specific variation in emergency presentation by sex, age and deprivation across 27 common and rarer cancers. Br J Cancer. 2015;112:S129–36. 25734396 10.1038/bjc.2015.52 PMC4385986

[68] Weiner AB , Keeter M‐K , Manjunath A , Meeks JJ . Discrepancies in staging, treatment, and delays to treatment may explain disparities in bladder cancer outcomes: an update from the National Cancer Data Base (2004–2013). Urol Oncol Semin Orig Investig. 2018;1(36):237.e9–237.e17. 10.1016/j.urolonc.2017.12.015 29338913

