# Supplementary material for: Systematic review of the association between socioeconomic status and bladder cancer survival with hospital type, comorbidities, and treatment delay as mediators
Source: BJUI Compass. 2021 Jan 7;2(3):140–58. doi: 10.1002/bco2.65 (PMC8988826; doi:10.1002/bco2.65)

# **Appendix**

**Protocol**

1. **Formulate review question(s)**

**PICOS Question formulation**

**P**opulation – Bladder cancer patients

**I**ntervention/Exposure – SES/hospital type/comorbidities/treatment delay

**C**omparison –

SES: High vs. low SES

Hospital type: academic vs. non-academic hospital, large vs small hospital volume

Comorbidities: Mild/moderate vs. severe, existence of certain comorbidities vs. absence of said comorbidity, high vs. low comorbidity index

Treatment delay: Short vs. long delay

**O**utcomes – survival/SES

**S**tudy designs – RCTs, observational studies and reviews, written in English only

*Question 1: In patients with bladder cancer, does having a higher or lower SES have an effect on survival?*

*Question 2: In patients with bladder cancer, does having a higher or lower SES have an effect on what hospital type the patient is treated at?*

*Question 3: In patients with bladder cancer, does the hospital type in which patients are treated have an effect on survival?*

*Question 4: In patients with bladder cancer, does having a higher or lower SES have an effect on the presence of comorbidities?*

*Question 5: In patients with bladder cancer, does the level of comorbidities have an effect on survival?*

*Question 6: In patients with bladder cancer, does having a higher or lower SES have an effect on whether the patient experiences a delay in treatment?*

1. **Define search criteria**

**Search criteria**

1. **SES and survival**

(bladder cancer.mp. or exp bladder cancer/) and (exp social class/ or socio$/ or deprivation.mp.) and (exp survival/ or exp mortality/ or outcome.mp.) [mp=ti, ab, hw, tn, ot, dm, mf, dv, kw, fx, dq, nm, kf, ox, px, rx, an, ui, sy]

1. **SES and hospital type**

exp bladder cancer/ and (exp social class/ or socio$/ or deprivation.mp.) and (hospital and size).mp.

1. **Hospital type and survival**

exp bladder cancer/ and (exp survival/ or exp mortality/ or outcome.mp.) and ((hospital and size) or centralization or centralisation).mp

1. **SES and CCI**

exp bladder cancer/ and (exp social class/ or socio$/ or deprivation.mp.) and (comorbidity.sh. or exp condition)

1. **CCI and survival**

exp bladder cancer/ and (comorbidity.sh. or exp condition) and (exp survival/ or exp mortality/ OR outcome)

1. **SES and treatment delay**

exp bladder cancer/ and (exp social class/ or socio$/) and (time or Delay).mp. [mp=ti, ab, hw, tn, ot, dm, mf, dv, kw, fx, dq, nm, kf, ox, px, rx, an, ui, sy]

**Databases to be searched:**

- Medline (PubMed)
- Ovid Gateway (Embase and Ovid)

1. **Define inclusion and exclusion criteria**

**Inclusion criteria**

- RCT, observational study or review
- Must include bladder cancer patients
- Written in the English language
- Published in or after year 2000
- Must report on correct outcome as specified by PICO criteria

**Exclusion criteria**

- No mention of bladder cancer patients
- Commentaries, author’s replies, supplements, editorials, abstracts only
- Incorrect outcome reported

Supplementary Table 1. Risk of bias for studies using the Risk of Bias In Non-randomised Studies - of Interventions (ROBINS-I) tool: SES and survival.

| Signalling questions | SES and survival | | | | | | | | | | | |
| --- | --- | --- | --- | --- | --- | --- | --- | --- | --- | --- | --- | --- |
|  | Lara et al. | Eberle et al. | Sloggett et al. | Klapheke et al. | Chien et al. | Begum et al. | Moran et al. | Coleman et al. | Belot et al. | Syriopoulou et al. | Sundquist et al. | Shack et al. |
| 1.1 Is there potential for confounding of the effect of intervention in this study? | Y | Y | Y | Y | Y | Y | Y | Y | Y | Y | Y | Y |
| If N/PN to 1.1: the study can be considered to be at low risk of bias due to confounding and no further signalling questions need be considered | - | - | - | - | - | - | - | - | - | - | - | - |
| If Y/PY to 1.1: determine whether there is a need to assess time-varying confounding: | - | - | - | - | - | - | - | - | - | - | - | - |
| 1.2. Was the analysis based on splitting participants’ follow up time according to intervention received? | N | N | N | N | N | N | N | N | N | N | N | N |
| If N/PN, answer questions relating to baseline confounding (1.4 to 1.6) If Y/PY, proceed to question 1.3. | - | - | - | - | - | - | - | - | - | - | - | - |
| 1.3. Were intervention discontinuations or switches likely to be related to factors that are prognostic for the outcome? | - | - | - | - | - | - | - | - | - | - | - | - |
| If N/PN, answer questions relating to baseline confounding (1.4 to 1.6) If Y/PY, answer questions relating to both baseline and time-varying confounding (1.7 and 1.8) | - | - | - | - | - | - | - | - | - | - | - | - |
| **Questions relating to baseline confounding only** |  |  |  |  |  |  |  |  |  |  |  |  |
| 1.4. Did the authors use an appropriate analysis method that controlled for all the important confounding domains? | Y | PN | N | Y | PN | Y | N | Y | Y | N | Y | Y |
| 1.5. If Y/PY to 1.4: Were confounding domains that were controlled for measured validly and reliably by the variables available in this study? | PY | - | - | Y | - | Y | - | Y | Y | - | Y | Y |
| 1.6. Did the authors control for any post-intervention variables that could have been affected by the intervention? | N | - | - | N | - | N | - | N | N | - | N | N |
| **Questions relating to baseline and time-varying confounding** |  |  |  |  |  |  |  |  |  |  |  |  |
| 1.7. Did the authors use an appropriate analysis method that adjusted for all the important confounding domains and for time-varying confounding? | - | - | - | - | - | - | - | - | - | - | - | - |
| 1.8. If Y/PY to 1.7: Were confounding domains that were adjusted for measured validly and reliably by the variables available in this study? | - | - | - | - | - | - | - | - | - | - | - | - |
| **Risk of bias judgement** | Low | Mod | Mod | Low | Mod | Low | Mod | Low | Low | Mod | Low | Low |
| 2.1. Was selection of participants into the study (or into the analysis) based on participant characteristics observed after the start of intervention? | N | PN | PN | N | N | N | N | N | N | N | N | N |
| If N/PN to 2.1: go to 2.4 |  |  |  |  |  |  |  |  |  |  |  |  |
| 2.2. If Y/PY to 2.1: Were the post-intervention variables that influenced selection likely to be associated with intervention? | - | - | - | - | - | - | - | - | - | - | - | - |
| 2.3 If Y/PY to 2.2: Were the post-intervention variables that influenced selection likely to be influenced by the outcome or a cause of the outcome? | - | - | - | - | - | - | - | - | - | - | - | - |
| 2.4. Do start of follow-up and start of intervention coincide for most participants? | Y | Y | Y | Y | PY | Y | PY | PY | PY | PY | PY | PY |
| 2.5. If Y/PY to 2.2 and 2.3, or N/PN to 2.4: Were adjustment techniques used that are likely to correct for the presence of selection biases? | - | - | - | - | - | - | - | - | - | - | - | - |
| **Risk of bias judgement** | Low | Low | Low | Low | Low | Low | Low | Low | Low | Low | Low | Low |
| 3.1 Were intervention groups clearly defined? | Y | Y | Y | Y | Y | Y | Y | Y | Y | Y | Y | Y |
| 3.2 Was the information used to define intervention groups recorded at the start of the intervention? | Y | Y | Y | Y | Y | Y | Y | Y | Y | Y | Y | Y |
| 3.3 Could classification of intervention status have been affected by knowledge of the outcome or risk of the outcome? | N | N | N | N | N | N | N | N | N | N | N | N |
| **Risk of bias judgement** | Low | Low | Low | Low | Low | Low | Low | Low | Low | Low | Low | Low |
| **If your aim for this study is to assess the effect of assignment to intervention, answer questions 4.1 and 4.2** |  |  |  |  |  |  |  |  |  |  |  |  |
| 4.1. Were there deviations from the intended intervention beyond what would be expected in usual practice? | PN | PN | PN | PN | PN | PN | PN | PN | PN | PN | PN | PN |
| 4.2. If Y/PY to 4.1: Were these deviations from intended intervention unbalanced between groups and likely to have affected the outcome? | - | - | - | - | - | - | - | - | - | - | - | - |
| **If your aim for this study is to assess the effect of starting and adhering to intervention, answer questions 4.3 to 4.6** |  |  |  |  |  |  |  |  |  |  |  |  |
| 4.3. Were important co-interventions balanced across intervention groups? | - | - | - | - | - | - | - | - | - | - | - | - |
| 4.4. Was the intervention implemented successfully for most participants? | - | - | - | - | - | - | - | - | - | - | - | - |
| 4.5. Did study participants adhere to the assigned intervention regimen? | - | - | - | - | - | - | - | - | - | - | - | - |
| 4.6. If N/PN to 4.3, 4.4 or 4.5: Was an appropriate analysis used to estimate the effect of starting and adhering to the intervention? | - | - | - | - | - | - | - | - | - | - | - | - |
| **Risk of bias judgement** | Low | Low | Low | Low | Low | Low | Low | Low | Low | Low | Low | Low |
| 5.1 Were outcome data available for all, or nearly all, participants? | Y | Y | Y | Y | PY | Y | PY | Y | Y | Y | Y | Y |
| 5.2 Were participants excluded due to missing data on intervention status? | N | N | Y | PN | N | N | PN | N | N | N | N | N |
| 5.3 Were participants excluded due to missing data on other variables needed for the analysis? | PN | N | N | PN | N | N | PN | N | N | N | N | N |
| 5.4 If PN/N to 5.1, or Y/PY to 5.2 or 5.3: Are the proportion of participants and reasons for missing data similar across interventions? | - | - | - | - | - | - | - | - | - | - | - | - |
| 5.5 If PN/N to 5.1, or Y/PY to 5.2 or 5.3: Is there evidence that results were robust to the presence of missing data? | - | - | - | - | - | - | - | - | - | - | - | - |
| **Risk of bias judgement** | Low | Low | Mod | Low | Low | Low | Low | Low | Low | Low | Low | Low |
| 6.1 Could the outcome measure have been influenced by knowledge of the intervention received? | N | N | N | N | N | N | N | N | N | N | N | N |
| 6.2 Were outcome assessors aware of the intervention received by study participants? | N | N | N | N | N | N | N | N | N | N | N | N |
| 6.3 Were the methods of outcome assessment comparable across intervention groups? | Y | Y | Y | Y | Y | Y | Y | Y | Y | Y | Y | Y |
| 6.4 Were any systematic errors in measurement of the outcome related to intervention received? | N | N | N | N | N | N | N | N | N | N | N | N |
| **Risk of bias judgement** | Low | Low | Low | Low | Low | Low | Low | Low | Low | Low | Low | Low |
| Is the reported effect estimate likely to be selected, on the basis of the results, from... |  |  |  |  |  |  |  |  |  |  |  |  |
| 7.1. ... multiple outcome measurements within the outcome domain? | N | N | N | N | N | N | N | N | N | N | N | N |
| 7.2 ... multiple analyses of the intervention-outcome relationship? | N | N | N | N | N | N | N | N | N | N | N | N |
| 7.3 ... different subgroups? | N | N | N | N | N | N | N | N | N | N | N | N |
| **Risk of bias judgement** | Low | Low | Low | Low | Low | Low | Low | Low | Low | Low | Low | Low |
|  |  |  |  |  |  |  |  |  |  |  |  |  |
| **Risk of bias judgement** | **Low** | **Mod** | **Mod** | **Low** | **Mod** | **Low** | **Mod** | **Low** | **Low** | **Mod** | **Low** | **Low** |

N= no; PN= probably no; Y = yes; PY= probably yes; N/A= not applicable. Mod= Moderate risk of bias

Supplementary Table 2. Risk of bias for studies using the Risk of Bias In Non-randomised Studies - of Interventions (ROBINS-I) tool: Hospital type and survival.

| Signalling questions | Hospital type and survival | | | | | | | | | | | | |
| --- | --- | --- | --- | --- | --- | --- | --- | --- | --- | --- | --- | --- | --- |
|  | Bajaj et al. | Goossens-Laan et al. | Mayer et al. | Birkmeyer et al. | Udovicich et al | Scarberry et al. | Hounsome et al. | Liedberg et al. | Afshar et al. | Leow et al. | McCabe et al. | de Vries et al. | Lieberman-Cribbin et al. |
| 1.1 Is there potential for confounding of the effect of intervention in this study? | Y | Y | Y | Y | Y | Y | Y | Y | Y | Y | N | Y | N |
| If N/PN to 1.1: the study can be considered to be at low risk of bias due to confounding and no further signalling questions need be considered | - | - | - | - | - | - | - | - | - | - | - | - | - |
| If Y/PY to 1.1: determine whether there is a need to assess time-varying confounding: | - | - | - | - | - | - | - | - | - | - | - | - | - |
| 1.2. Was the analysis based on splitting participants’ follow up time according to intervention received? | N | N | N | N | N | N | N | N | N | N | - | N | - |
| If N/PN, answer questions relating to baseline confounding (1.4 to 1.6) If Y/PY, proceed to question 1.3. | - | - | - | - | - | - | - | - | - | - | - | - | - |
| 1.3. Were intervention discontinuations or switches likely to be related to factors that are prognostic for the outcome? | - | - | - | - | - | - | - | - | - | - | - | - | - |
| If N/PN, answer questions relating to baseline confounding (1.4 to 1.6) If Y/PY, answer questions relating to both baseline and time-varying confounding (1.7 and 1.8) | - | - | - | - | - | - | - | - | - | - | - | - | - |
| **Questions relating to baseline confounding only** |  |  |  |  |  |  |  |  |  |  |  |  |  |
| 1.4. Did the authors use an appropriate analysis method that controlled for all the important confounding domains? | Y | Y | Y | Y | Y | Y | Y | Y | Y | Y | - | Y | - |
| 1.5. If Y/PY to 1.4: Were confounding domains that were controlled for measured validly and reliably by the variables available in this study? | Y | Y | Y | Y | Y | Y | Y | Y | Y | Y | - | Y | - |
| 1.6. Did the authors control for any post-intervention variables that could have been affected by the intervention? | N | N | N | N | N | N | N | N | N | N | - | N | - |
| **Questions relating to baseline and time-varying confounding** |  |  |  |  |  |  |  |  |  |  |  |  |  |
| 1.7. Did the authors use an appropriate analysis method that adjusted for all the important confounding domains and for time-varying confounding? | - | - | - | - | - | - | - | - | - | - | - | - | - |
| 1.8. If Y/PY to 1.7: Were confounding domains that were adjusted for measured validly and reliably by the variables available in this study? | - | - | - | - | - | - | - | - | - | - | - | - | - |
| **Risk of bias judgement** | Low | Low | Low | Low | Low | Low | Low | Low | Low | Low | - | Low | - |
| 2.1. Was selection of participants into the study (or into the analysis) based on participant characteristics observed after the start of intervention? | N | N | N | N | N | N | N | N | N | N | - | N | - |
| If N/PN to 2.1: go to 2.4 |  |  |  |  |  |  |  |  |  |  |  |  |  |
| 2.2. If Y/PY to 2.1: Were the post-intervention variables that influenced selection likely to be associated with intervention? | - | - | - | - | - | - | - | - | - | - | - | - | - |
| 2.3 If Y/PY to 2.2: Were the post-intervention variables that influenced selection likely to be influenced by the outcome or a cause of the outcome? | - | - | - | - | - | - | - | - | - | - | - | - | - |
| 2.4. Do start of follow-up and start of intervention coincide for most participants? | Y | PY | PY | PY | Y | Y | Y | Y | PY | Y | - | PY | - |
| 2.5. If Y/PY to 2.2 and 2.3, or N/PN to 2.4: Were adjustment techniques used that are likely to correct for the presence of selection biases? | - | - | - | - | - | - | - | - | - | - | - | - | - |
| **Risk of bias judgement** | Low | Low | Low | Low | Low | Low | Low | Low | Low | Low | - | Low | - |
| 3.1 Were intervention groups clearly defined? | Y | Y | Y | Y | Y | Y | Y | Y | Y | Y | - | Y | - |
| 3.2 Was the information used to define intervention groups recorded at the start of the intervention? | Y | Y | Y | Y | Y | Y | PY | Y | Y | Y | - | Y | - |
| 3.3 Could classification of intervention status have been affected by knowledge of the outcome or risk of the outcome? | N | N | N | N | N | N | N | N | N | N | - |  | - |
| **Risk of bias judgement** | Low | Low | Low | Low | Low | Low | Low | Low | Low | Low | - |  | - |
| **If your aim for this study is to assess the effect of assignment to intervention, answer questions 4.1 and 4.2** |  |  |  |  |  |  |  |  |  |  |  |  |  |
| 4.1. Were there deviations from the intended intervention beyond what would be expected in usual practice? | PN | PN | PN | PN | N | N | N | N | N | N | - |  | - |
| 4.2. If Y/PY to 4.1: Were these deviations from intended intervention unbalanced between groups and likely to have affected the outcome? | - | - | - | - | - | - | - | - | - | - | - | - | - |
| **If your aim for this study is to assess the effect of starting and adhering to intervention, answer questions 4.3 to 4.6** |  |  |  |  |  |  |  |  |  |  |  |  |  |
| 4.3. Were important co-interventions balanced across intervention groups? | - | - | - | - | - | - | - | - | - | - | - | - | - |
| 4.4. Was the intervention implemented successfully for most participants? | - | - | - | - | - | - | - | - | - | - | - | - | - |
| 4.5. Did study participants adhere to the assigned intervention regimen? | - | - | - | - | - | - | - | - | - | - | - | - | - |
| 4.6. If N/PN to 4.3, 4.4 or 4.5: Was an appropriate analysis used to estimate the effect of starting and adhering to the intervention? | - | - | - | - | - | - | - | - | - | - | - | - | - |
| **Risk of bias judgement** | Low | Low | Low | Low | Low | Low | Low | Low | Low | Low | - | Low | - |
| 5.1 Were outcome data available for all, or nearly all, participants? | Y | Y | Y | Y | Y | Y | Y | Y | Y | Y | - | Y | - |
| 5.2 Were participants excluded due to missing data on intervention status? | N | N | N | N | N | N | N | N | N | N | - | N | - |
| 5.3 Were participants excluded due to missing data on other variables needed for the analysis? | N | N | N | N | N | PN | PN | PN | Y | N | - | N | - |
| 5.4 If PN/N to 5.1, or Y/PY to 5.2 or 5.3: Are the proportion of participants and reasons for missing data similar across interventions? | - | - | - | - | - | - | - | - | PY | - | - | - | - |
| 5.5 If PN/N to 5.1, or Y/PY to 5.2 or 5.3: Is there evidence that results were robust to the presence of missing data? | - | - | - | - | - | - | - | - | Y | - | - | - | - |
| **Risk of bias judgement** | Low | Low | Low | Low | Low | Low | Low | Low | Low | Low | - | Low | - |
| 6.1 Could the outcome measure have been influenced by knowledge of the intervention received? | N | N | N | N | N | N | N | N | N | N | - | N | - |
| 6.2 Were outcome assessors aware of the intervention received by study participants? | N | N | N | N | N | N | N | N | N | N | - | N | - |
| 6.3 Were the methods of outcome assessment comparable across intervention groups? | Y | Y | Y | Y | Y | Y | Y | Y | Y | Y | - | Y | - |
| 6.4 Were any systematic errors in measurement of the outcome related to intervention received? | N | N | N | N | N | N | N | N | N | N | - | N | - |
| **Risk of bias judgement** | Low | Low | Low | Low | Low | Low | Low | Low | Low | Low | - | Low | - |
| Is the reported effect estimate likely to be selected, on the basis of the results, from... |  |  |  |  |  |  |  |  |  |  |  |  |  |
| 7.1. ... multiple outcome measurements within the outcome domain? | N | N | N | N | N | N | N | N | N | N | - | N | - |
| 7.2 ... multiple analyses of the intervention-outcome relationship? | N | N | N | N | N | N | N | N | N | N | - | N | - |
| 7.3 ... different subgroups? | N | N | N | N | N | N | N | N | N | N | - | N | - |
| **Risk of bias judgement** | Low | Low | Low | Low | Low | Low | Low | Low | Low | Low | - | Low | - |
|  |  |  |  |  |  |  |  |  |  |  |  |  |  |
| **Risk of bias judgement** | Low | Low | Low | Low | Low | Low | Low | Low | Low | Low | Low | Low | Low |

N= no; PN= probably no; Y = yes; PY= probably yes.

Supplementary Table 3. Risk of bias for studies using the Risk of Bias In Non-randomised Studies - of Interventions (ROBINS-I) tool: Comorbidities and survival.

| Signalling questions | *Comorbidities and Survival* | | | | | | | | | | | | | | | | | |
| --- | --- | --- | --- | --- | --- | --- | --- | --- | --- | --- | --- | --- | --- | --- | --- | --- | --- | --- |
|  | Pereira et al | Racioppi et al. | Froehner et al | Johnson et al. | Dell'Oglio et al. | Sarfati et al. | Li et al. | Dybowski et al. | Goossens-Laan et al. | Mayr et al. | Mayr et al. | Lund et al. | Ha and Chang | Koppie et al. | Megwalu et al. | Zhu et al | Miller et al. | Boorjian et al. |
| 1.1 Is there potential for confounding of the effect of intervention in this study? | Y | Y | Y | Y | Y | Y | Y | Y | Y | Y | Y | Y | Y | Y | Y | Y | Y | Y |
| If N/PN to 1.1: the study can be considered to be at low risk of bias due to confounding and no further signalling questions need be considered | - | - | - | - | - | - | - | - | - | - | - | - | - | - | - | - | - | - |
| If Y/PY to 1.1: determine whether there is a need to assess time-varying confounding: | - | - | - | - | - | - | - | - | - | - | - | - | - | - | - | - | - | - |
| 1.2. Was the analysis based on splitting participants’ follow up time according to intervention received? | N | N | N | N | N | N | N | N | N | N | N | N | N | N | N | N | N | N |
| If N/PN, answer questions relating to baseline confounding (1.4 to 1.6) If Y/PY, proceed to question 1.3. | - | - | - | - | - | - | - | - | - | - | - | - | - | - | - | - | - | - |
| 1.3. Were intervention discontinuations or switches likely to be related to factors that are prognostic for the outcome? | - | - | - | - | - | - | - | - | - | - | - | - | - | - | - | - | - | - |
| If N/PN, answer questions relating to baseline confounding (1.4 to 1.6) If Y/PY, answer questions relating to both baseline and time-varying confounding (1.7 and 1.8) | - | - | - | - | - | - | - | - | - | - | - | - | - | - | - | - | - | - |
| **Questions relating to baseline confounding only** |  |  |  |  |  |  |  |  |  |  |  |  |  |  |  |  |  |  |
| 1.4. Did the authors use an appropriate analysis method that controlled for all the important confounding domains? | Y | N | Y | Y | Y | N | Y | Y | Y | Y | Y | N | Y | Y | Y | Y | Y | PN |
| 1.5. If Y/PY to 1.4: Were confounding domains that were controlled for measured validly and reliably by the variables available in this study? | Y | - | Y | Y | Y | Y | Y | Y | Y | y | y | - | y | Y | Y | Y | Y | - |
| 1.6. Did the authors control for any post-intervention variables that could have been affected by the intervention? | N | - | N | N | N | N | N | N | N | N | N | - | N | N | N | N | N | - |
| **Questions relating to baseline and time-varying confounding** |  |  |  |  |  |  |  |  |  |  |  |  |  |  |  |  |  |  |
| 1.7. Did the authors use an appropriate analysis method that adjusted for all the important confounding domains and for time-varying confounding? | - | - | - | - | - | - | - | - | - | - | - | - | - | - | - | - | - | - |
| 1.8. If Y/PY to 1.7: Were confounding domains that were adjusted for measured validly and reliably by the variables available in this study? | - | - | - | - | - | - | - | - | - | - | - | - | - | - | - | - | - | - |
| **Risk of bias judgement** | Low | Mod | Low | Low | Low | Mod | Low | Low | Low | Low | Low | Mod | Low | Low | Low | Low | Low | Mod |
| 2.1. Was selection of participants into the study (or into the analysis) based on participant characteristics observed after the start of intervention? | N | N | N | N | N | N | N | N | N | N | N | N | N | N | N | N | N | PN |
| If N/PN to 2.1: go to 2.4 |  |  |  |  |  |  |  |  |  |  |  |  |  |  |  |  |  |  |
| 2.2. If Y/PY to 2.1: Were the post-intervention variables that influenced selection likely to be associated with intervention? | - | - | - | - | - | - | - | - | - | - | - | - | - | - | - | - | - | - |
| 2.3 If Y/PY to 2.2: Were the post-intervention variables that influenced selection likely to be influenced by the outcome or a cause of the outcome? | - | - | - | - | - | - | - | - | - | - | - | - | - | - | - | - | - | - |
| 2.4. Do start of follow-up and start of intervention coincide for most participants? | Y | Y | Y | Y | Y | Y | Y | Y | Y | Y | Y | Y | Y | Y | Y | Y | Y | PY |
| 2.5. If Y/PY to 2.2 and 2.3, or N/PN to 2.4: Were adjustment techniques used that are likely to correct for the presence of selection biases? | - | - | - | - | - | - | - | - | - | - | - | - | - | - | - | - | - | - |
| **Risk of bias judgement** | Low | Low | Low | Low | Low | Low | Low | Low | Low | Low | Low | Low | Low | Low | Low | Low | Low | Low |
| 3.1 Were intervention groups clearly defined? | Y | Y | Y | Y | Y | Y | Y | Y | Y | Y | Y | Y | Y | Y | Y | Y | Y | Y |
| 3.2 Was the information used to define intervention groups recorded at the start of the intervention? | Y | Y | Y | Y | Y | Y | Y | Y | Y | Y | Y | Y | Y | Y | Y | Y | Y | Y |
| 3.3 Could classification of intervention status have been affected by knowledge of the outcome or risk of the outcome? | N | N | N | N | N | N | N | N | N | N | N | N | N | N | N | N | N | N |
| **Risk of bias judgement** | Low | Low | Low | Low | Low | Low | Low | Low | Low | Low | Low | Low | Low | Low | Low | Low | Low | Low |
| **If your aim for this study is to assess the effect of assignment to intervention, answer questions 4.1 and 4.2** |  |  |  |  |  |  |  |  |  |  |  |  |  |  |  |  |  |  |
| 4.1. Were there deviations from the intended intervention beyond what would be expected in usual practice? | PN | PN | PN | PN | PN | PN | PN | PN | PN | PN | PN | PN | PN | PN | PN | PN | PN | PN |
| 4.2. If Y/PY to 4.1: Were these deviations from intended intervention unbalanced between groups and likely to have affected the outcome? | - | - | - | - | - | - | - | - | - | - | - | - | - | - | - | - | - | - |
| **If your aim for this study is to assess the effect of starting and adhering to intervention, answer questions 4.3 to 4.6** |  |  |  |  |  |  |  |  |  |  |  |  |  |  |  |  |  |  |
| 4.3. Were important co-interventions balanced across intervention groups? | - | - | - | - | - | - | - | - | - | - | - | - | - | - | - | - | - | - |
| 4.4. Was the intervention implemented successfully for most participants? | - | - | - | - | - | - | - | - | - | - | - | - | - | - | - | - | - | - |
| 4.5. Did study participants adhere to the assigned intervention regimen? | - | - | - | - | - | - | - | - | - | - | - | - | - | - | - | - | - | - |
| 4.6. If N/PN to 4.3, 4.4 or 4.5: Was an appropriate analysis used to estimate the effect of starting and adhering to the intervention? | - | - | - | - | - | - | - | - | - | - | - | - | - | - | - | - | - | - |
| **Risk of bias judgement** | Low | Low | Low | Low | Low | Low | Low | Low | Low | Low | Low | Low | Low | Low | Low | Low | Low | Low |
| 5.1 Were outcome data available for all, or nearly all, participants? | PY | PY | PY | PY | PY | PY | PY | PY | PY | PY | PY | PY | PY | PY | PY | Y | Y | Y |
| 5.2 Were participants excluded due to missing data on intervention status? | N | N | N | N | N | N | N | N | N | N | N | N | N | N | N | N | N | N |
| 5.3 Were participants excluded due to missing data on other variables needed for the analysis? | N | N | N | N | N | N | N | N | N | N | N | N | N | N | N | N | N | N |
| 5.4 If PN/N to 5.1, or Y/PY to 5.2 or 5.3: Are the proportion of participants and reasons for missing data similar across interventions? | - | - | - | - | - | - | - | - | - | - | - | - | - | - | - | - | - | - |
| 5.5 If PN/N to 5.1, or Y/PY to 5.2 or 5.3: Is there evidence that results were robust to the presence of missing data? | - | - | - | - | - | - | - | - | - | - | - | - | - | - | - | - | - | - |
| **Risk of bias judgement** | Low | Low | Low | Low | Low | Low | Low | Low | Low | Low | Low | Low | Low | Low | Low | Low | Low | Low |
| 6.1 Could the outcome measure have been influenced by knowledge of the intervention received? | N | N | N | N | N | N | N | N | N | N | N | N | N | N | N | N | N | N |
| 6.2 Were outcome assessors aware of the intervention received by study participants? | N | N | N | N | N | N | N | N | N | N | N | N | N | N | N | N | N | N |
| 6.3 Were the methods of outcome assessment comparable across intervention groups? | Y | Y | Y | Y | Y | Y | Y | Y | Y | Y | Y | Y | Y | Y | Y | Y | Y | Y |
| 6.4 Were any systematic errors in measurement of the outcome related to intervention received? | N | N | N | N | N | N | N | N | N | N | N | N | N | N | N | N | N | N |
| **Risk of bias judgement** | Low | Low | Low | Low | Low | Low | Low | Low | Low | Low | Low | Low | Low | Low | Low | Low | Low | Low |
| Is the reported effect estimate likely to be selected, on the basis of the results, from... |  |  |  |  |  |  |  |  |  |  |  |  |  |  |  |  |  |  |
| 7.1. ... multiple outcome measurements within the outcome domain? | N | N | N | N | N | N | N | N | N | N | N | N | N | N | N | N | N | N |
| 7.2 ... multiple analyses of the intervention-outcome relationship? | N | N | N | N | N | N | N | N | N | N | N | N | N | N | N | N | N | N |
| 7.3 ... different subgroups? | N | N | N | N | N | N | N | N | N | N | N | N | N | N | N | N | N | N |
| **Risk of bias judgement** | Low | Low | Low | Low | Low | Low | Low | Low | Low | Low | Low | Low | Low | Low | Low | Low | Low | Low |
|  |  |  |  |  |  |  |  |  |  |  |  |  |  |  |  |  |  |  |
| **Risk of bias judgement** | **Low** | **Mod** | **Low** | **Low** | **Low** | **Mod** | **Low** | **Low** | **Low** | **Low** | **Low** | **Mod** | **Low** | **Low** | **Low** | **Low** | **Low** | **Mod** |

N= no; PN= probably no; Y = yes; PY= probably yes; N/A= not applicable. Mod= Moderate risk of bias

Supplementary Figure 1. Assessment of systematic reviews according to AMSTAR criteria.


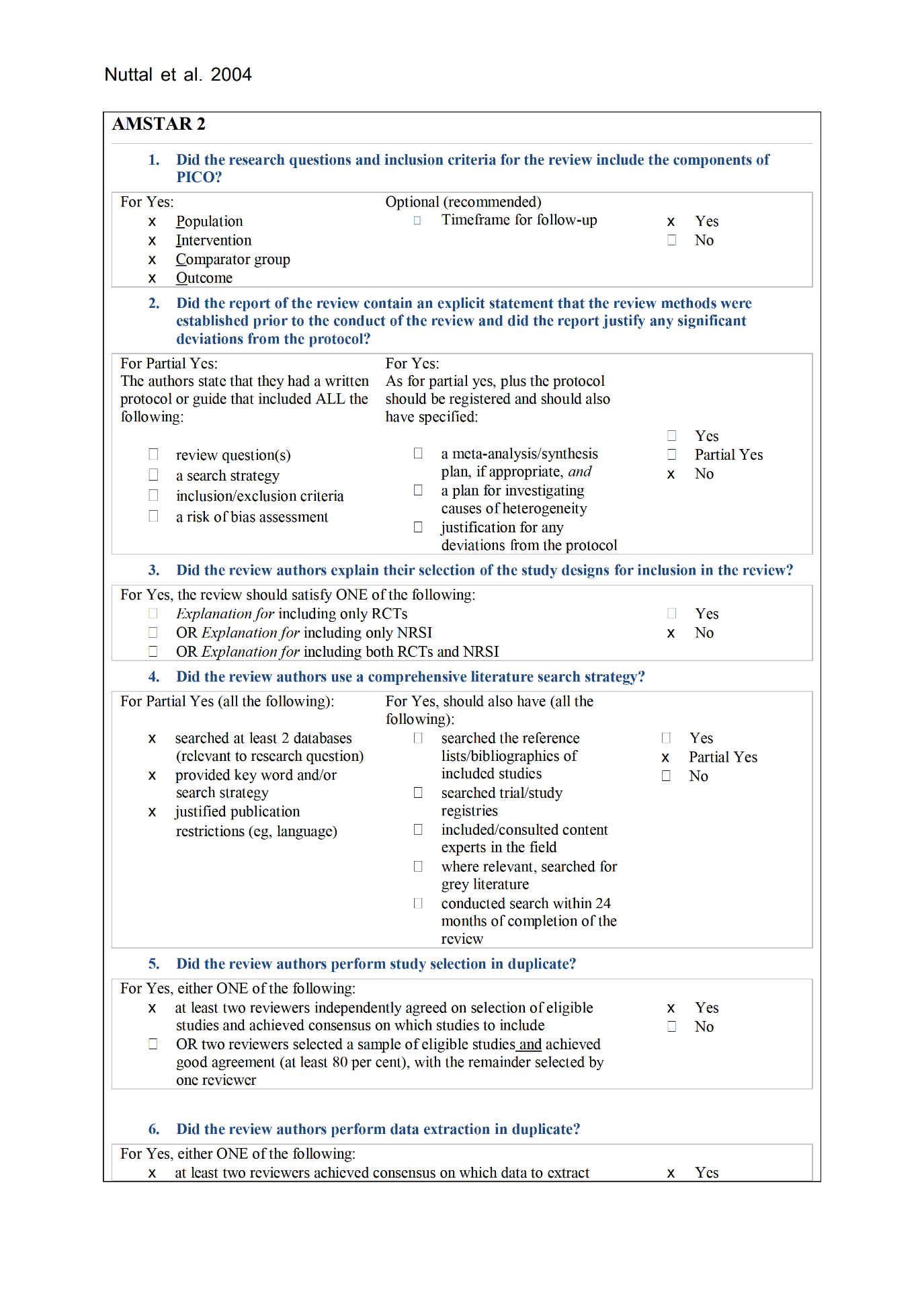


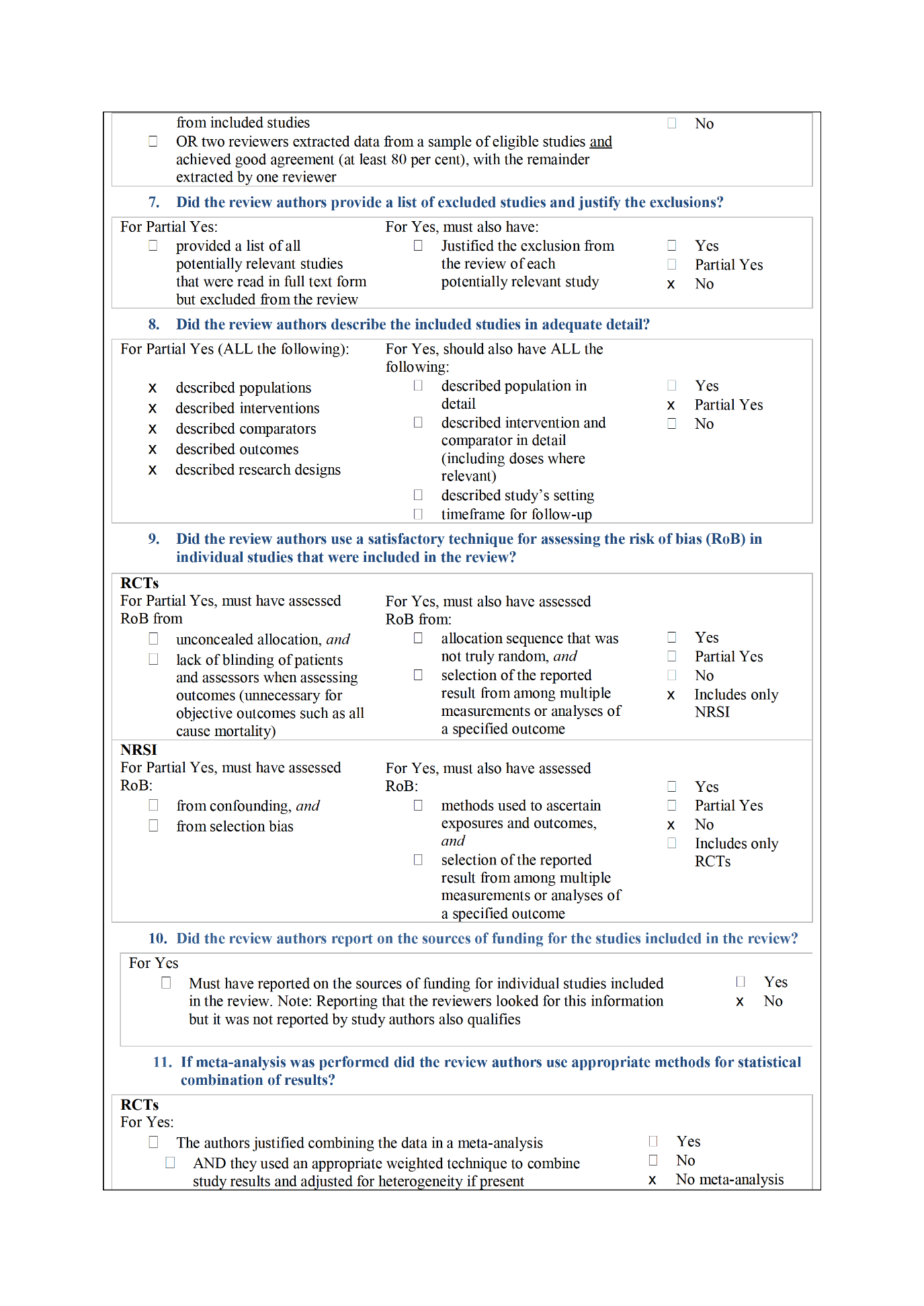


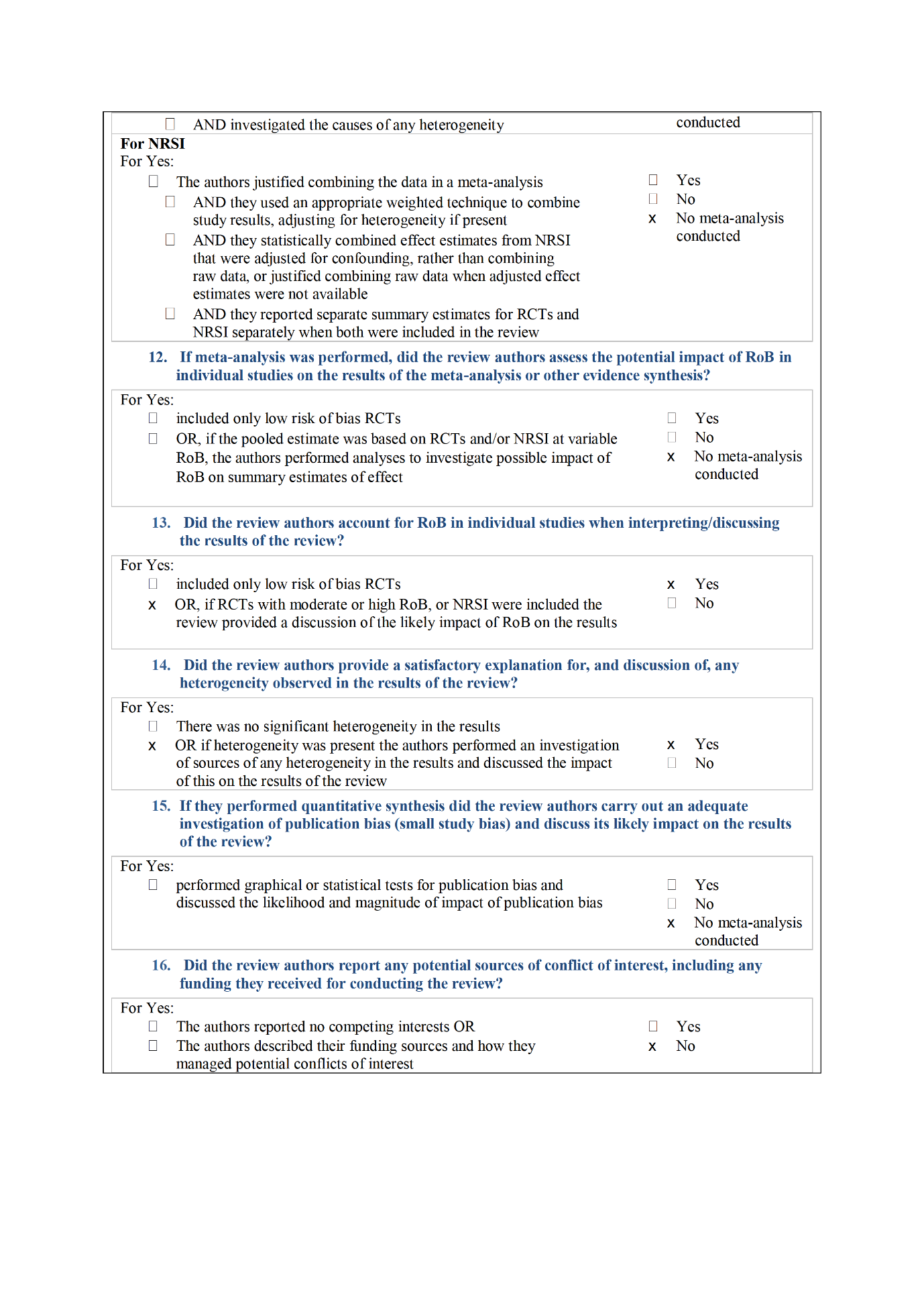


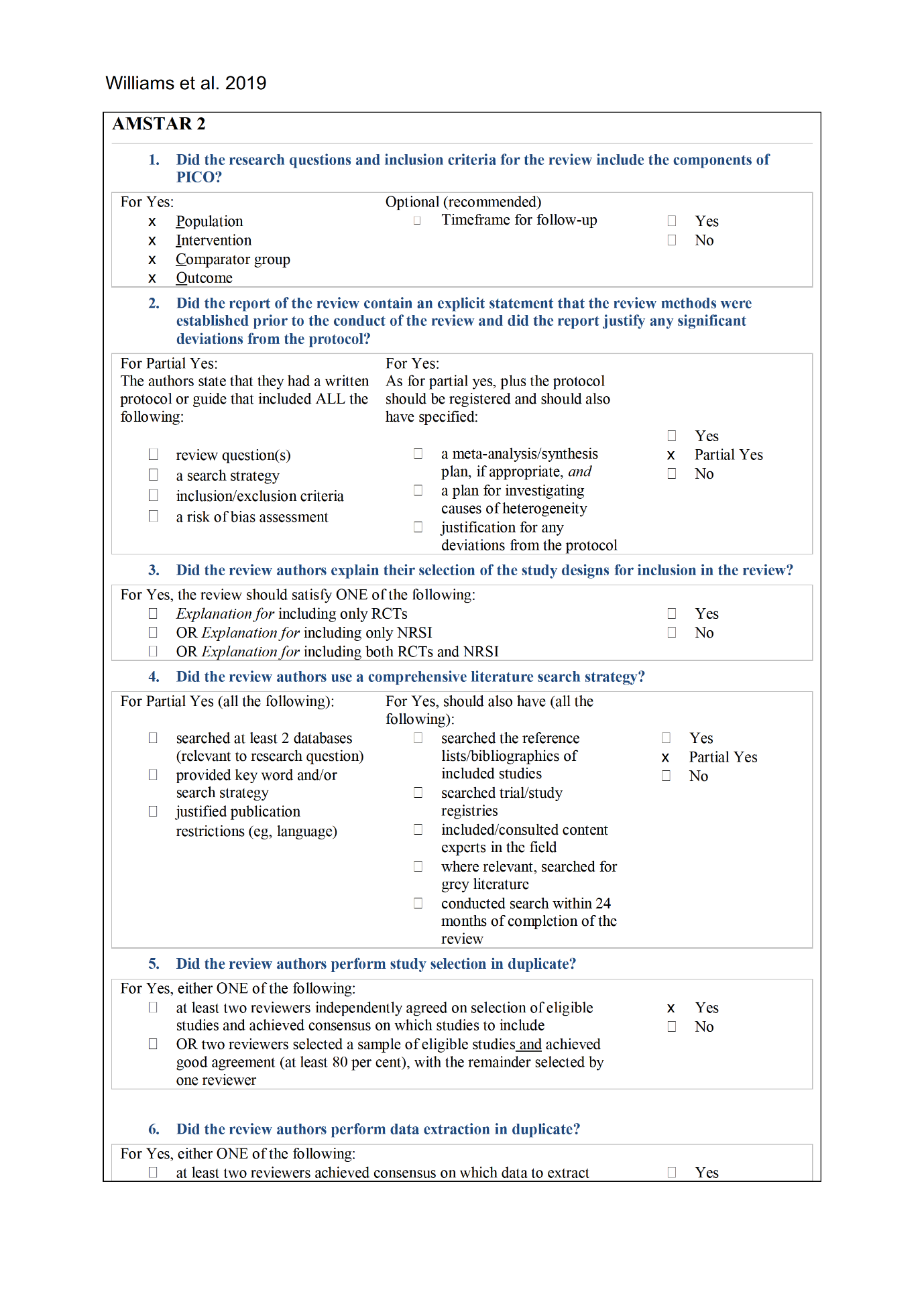


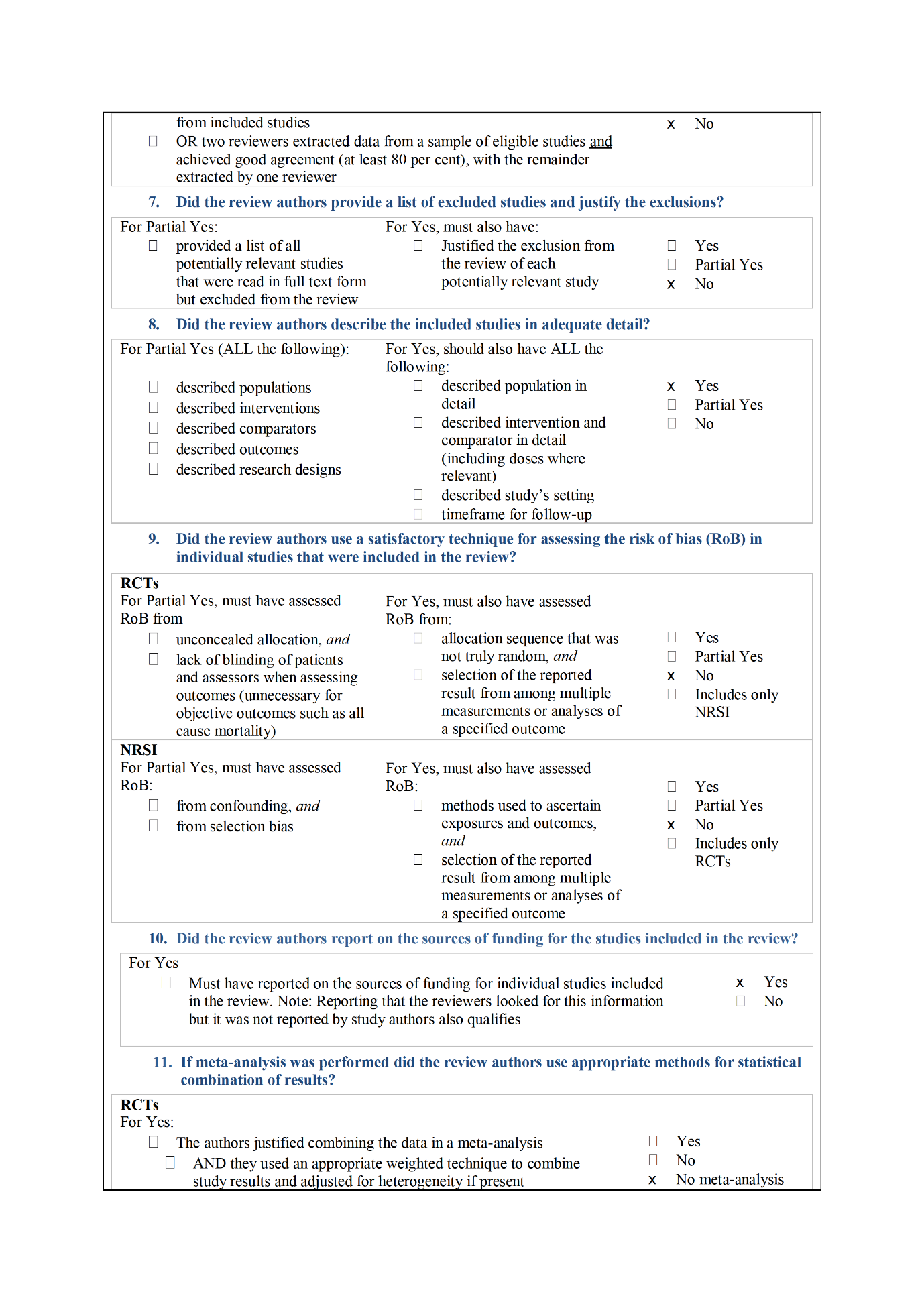


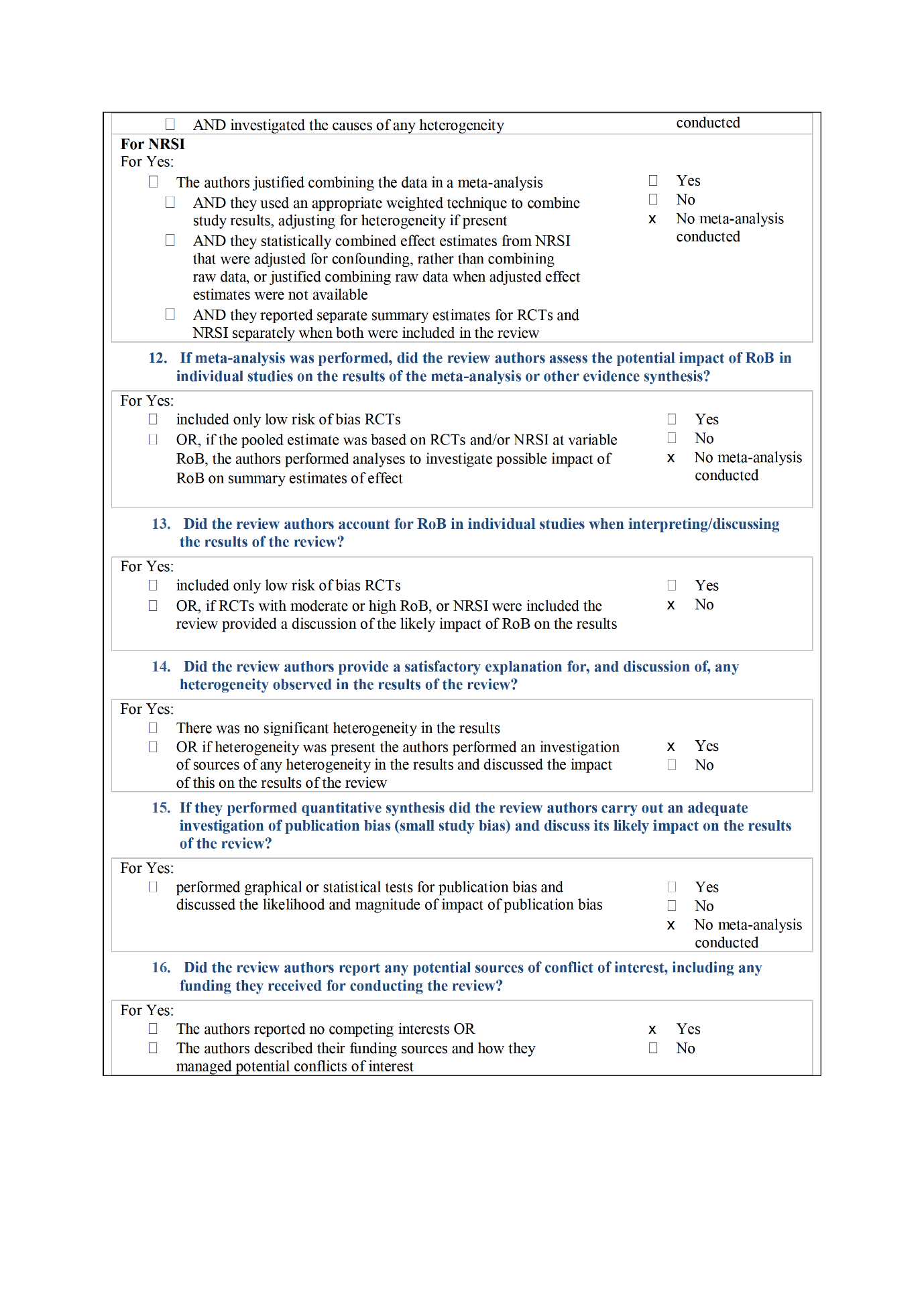


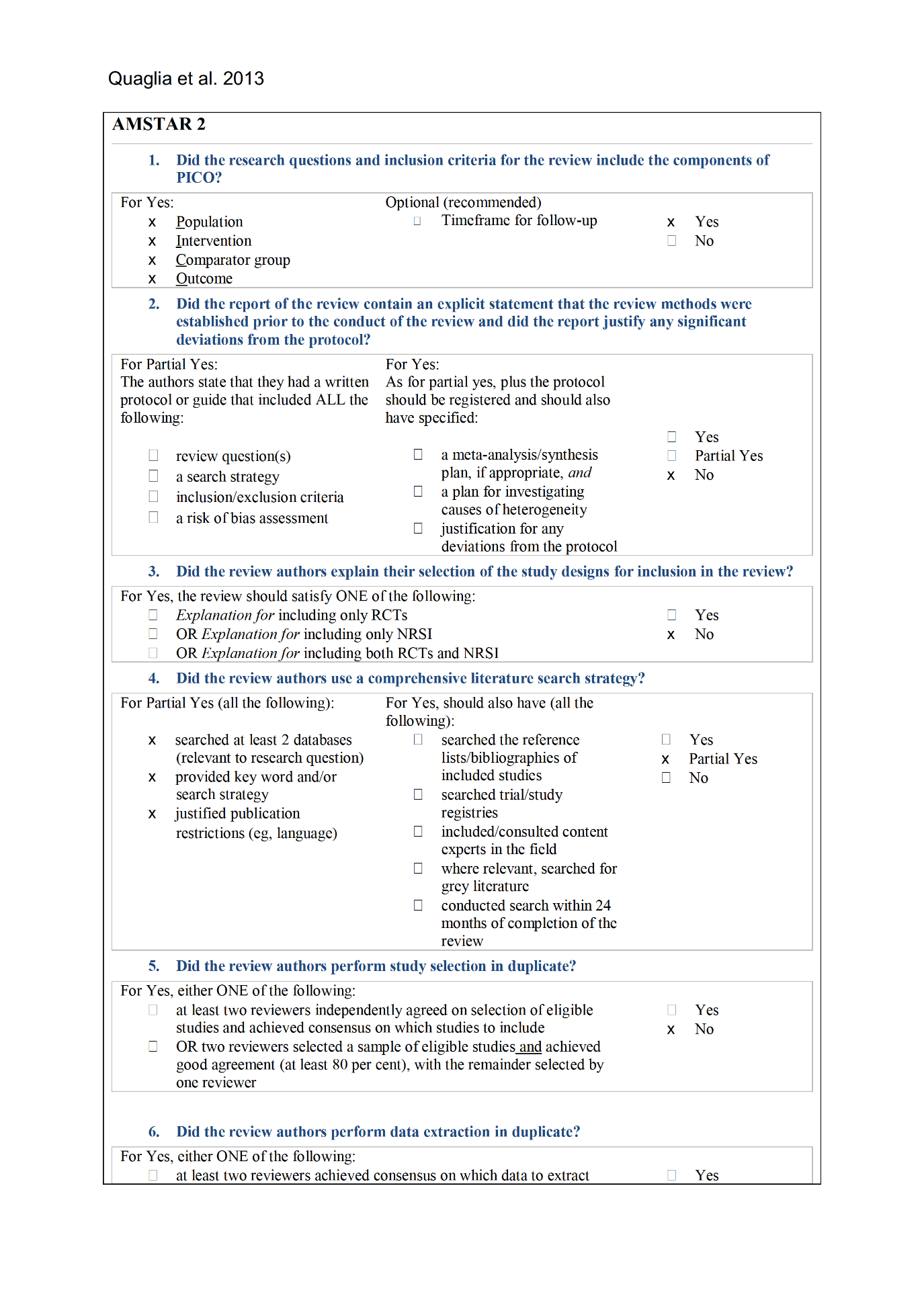


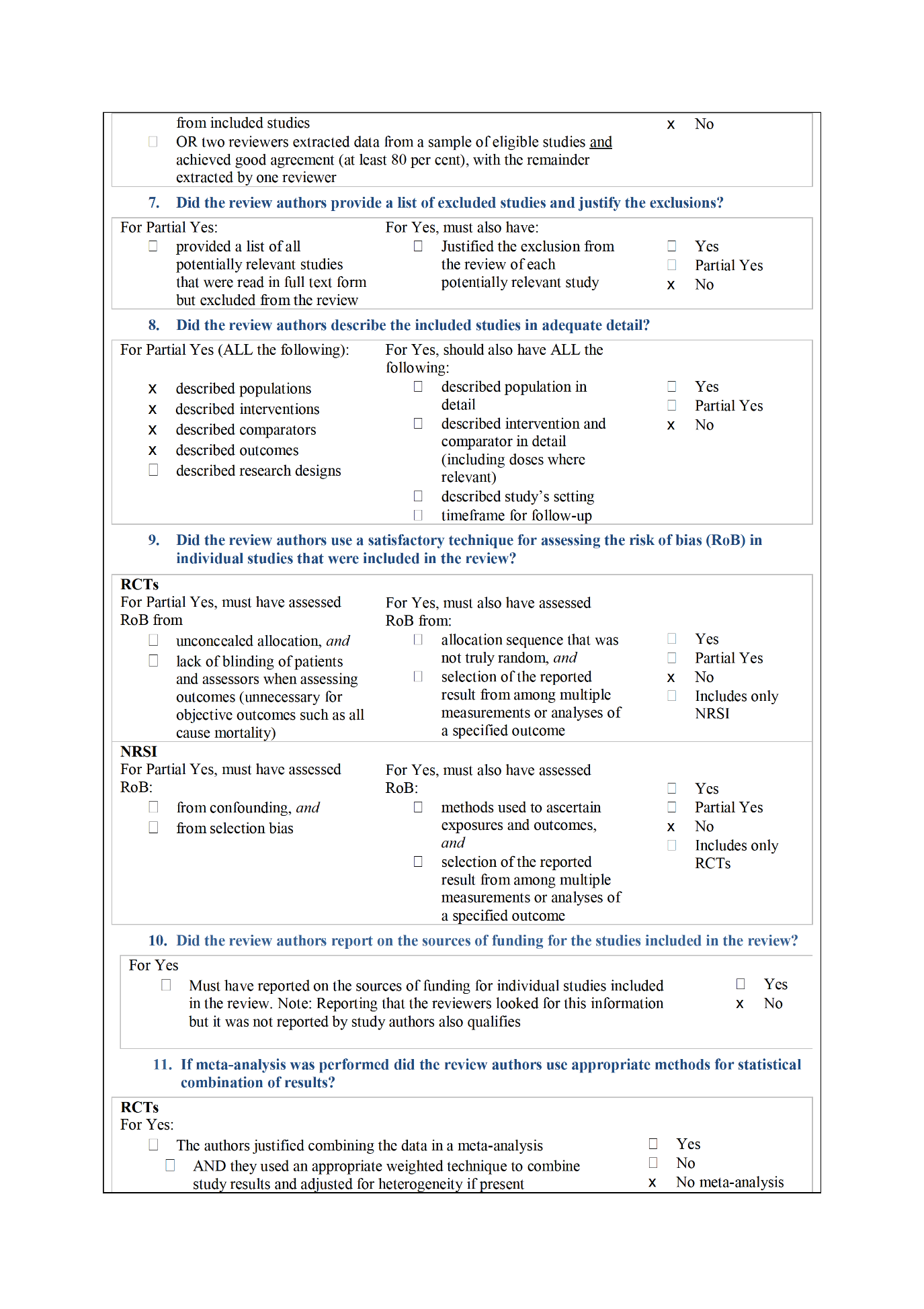


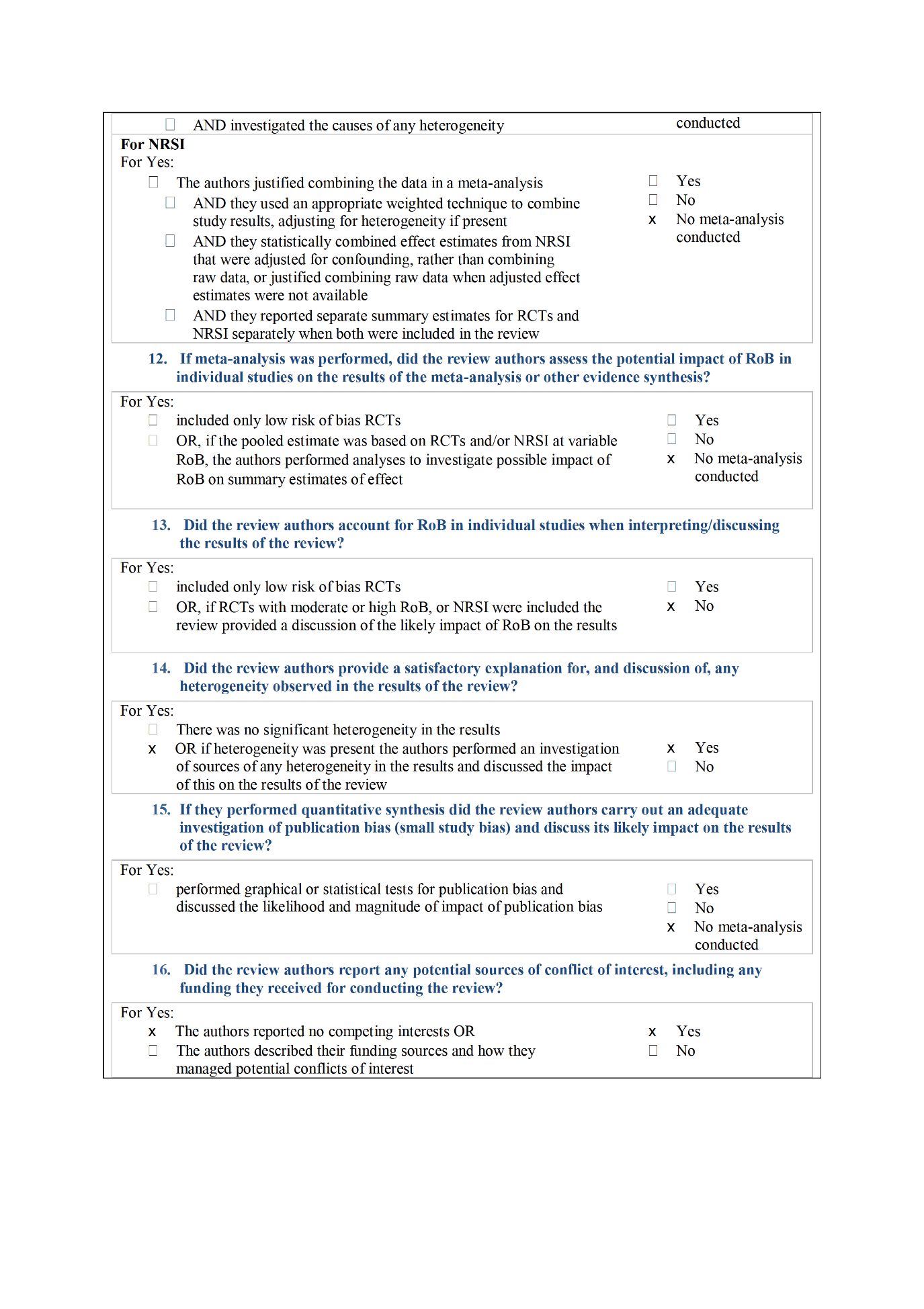


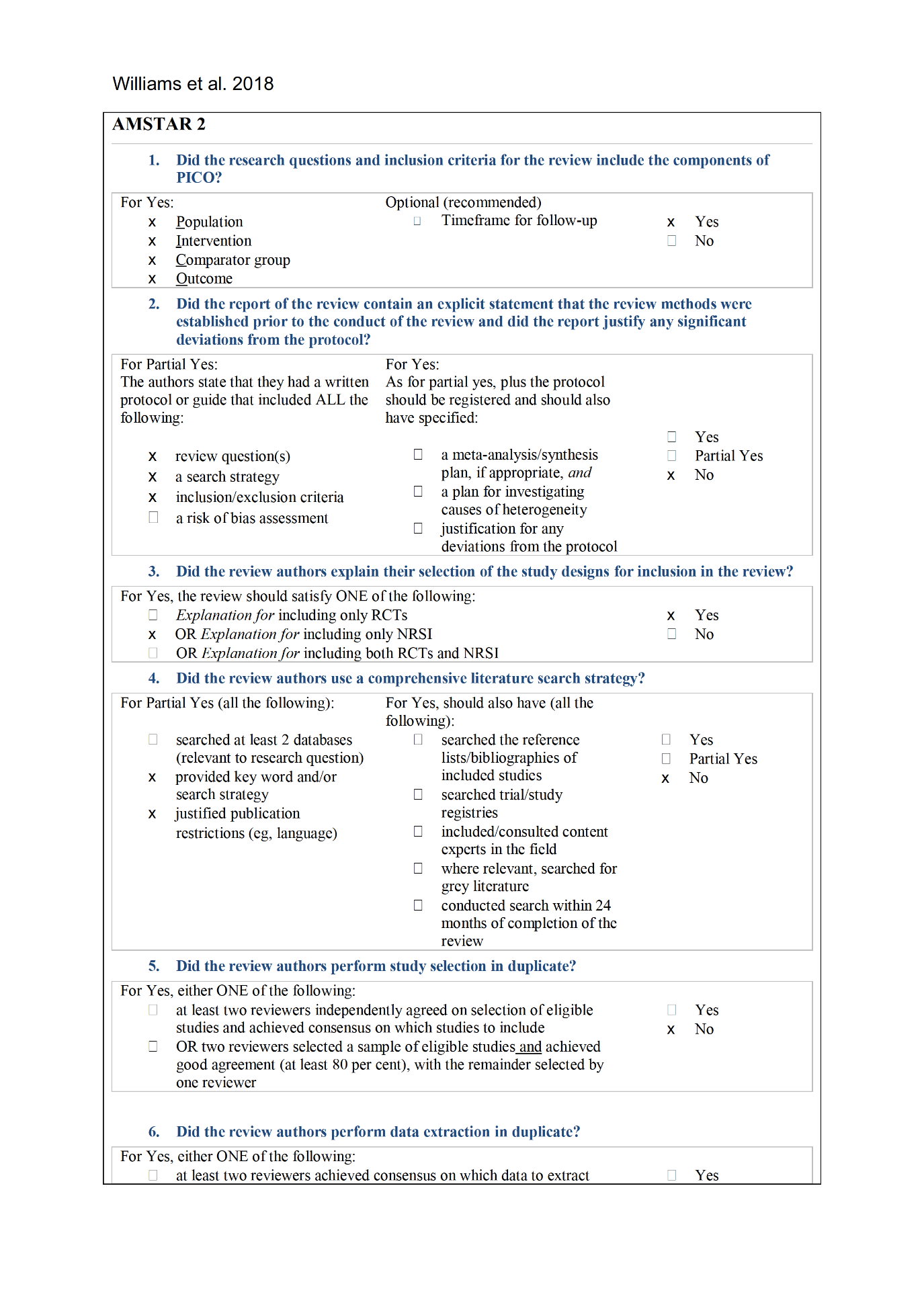


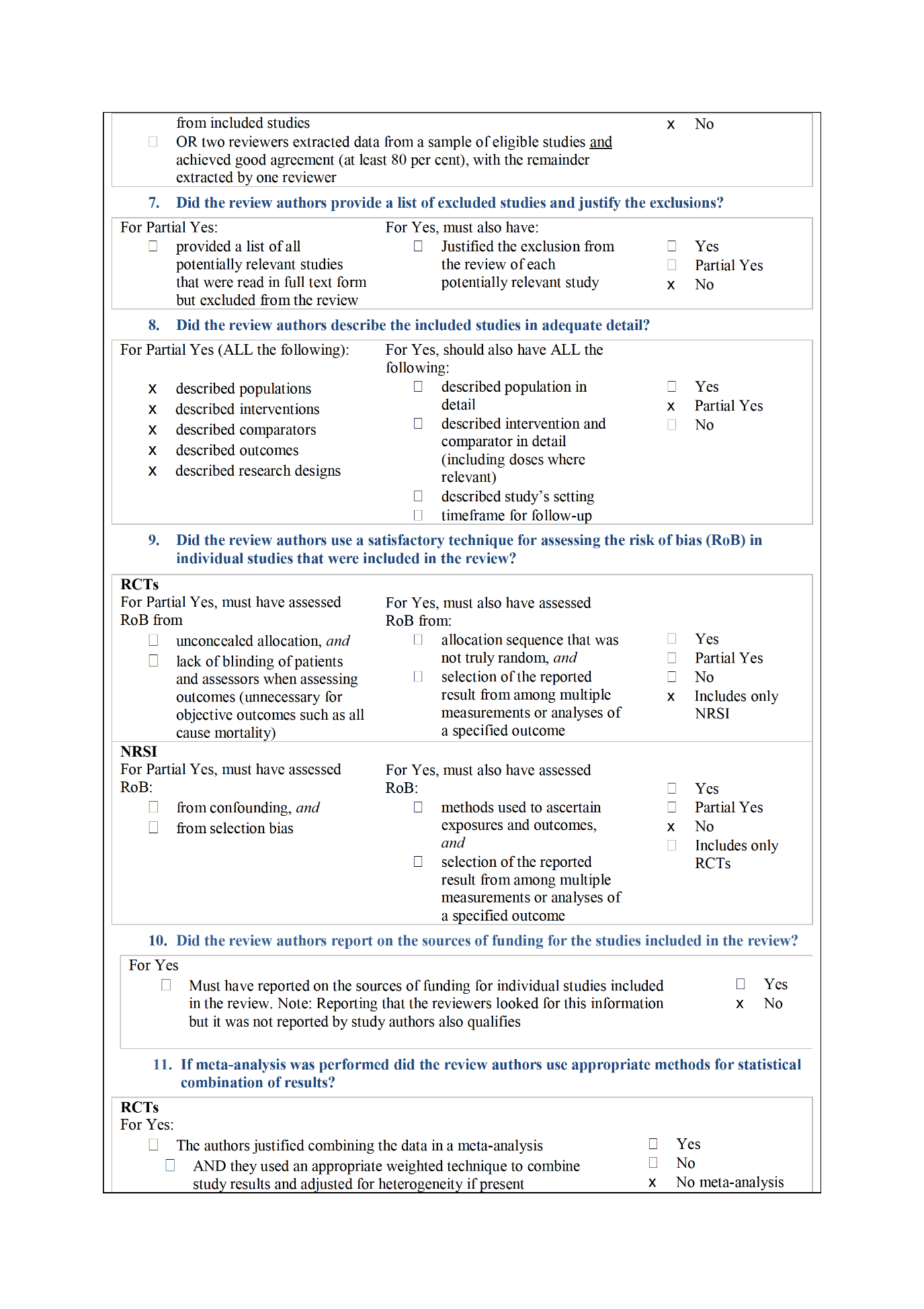


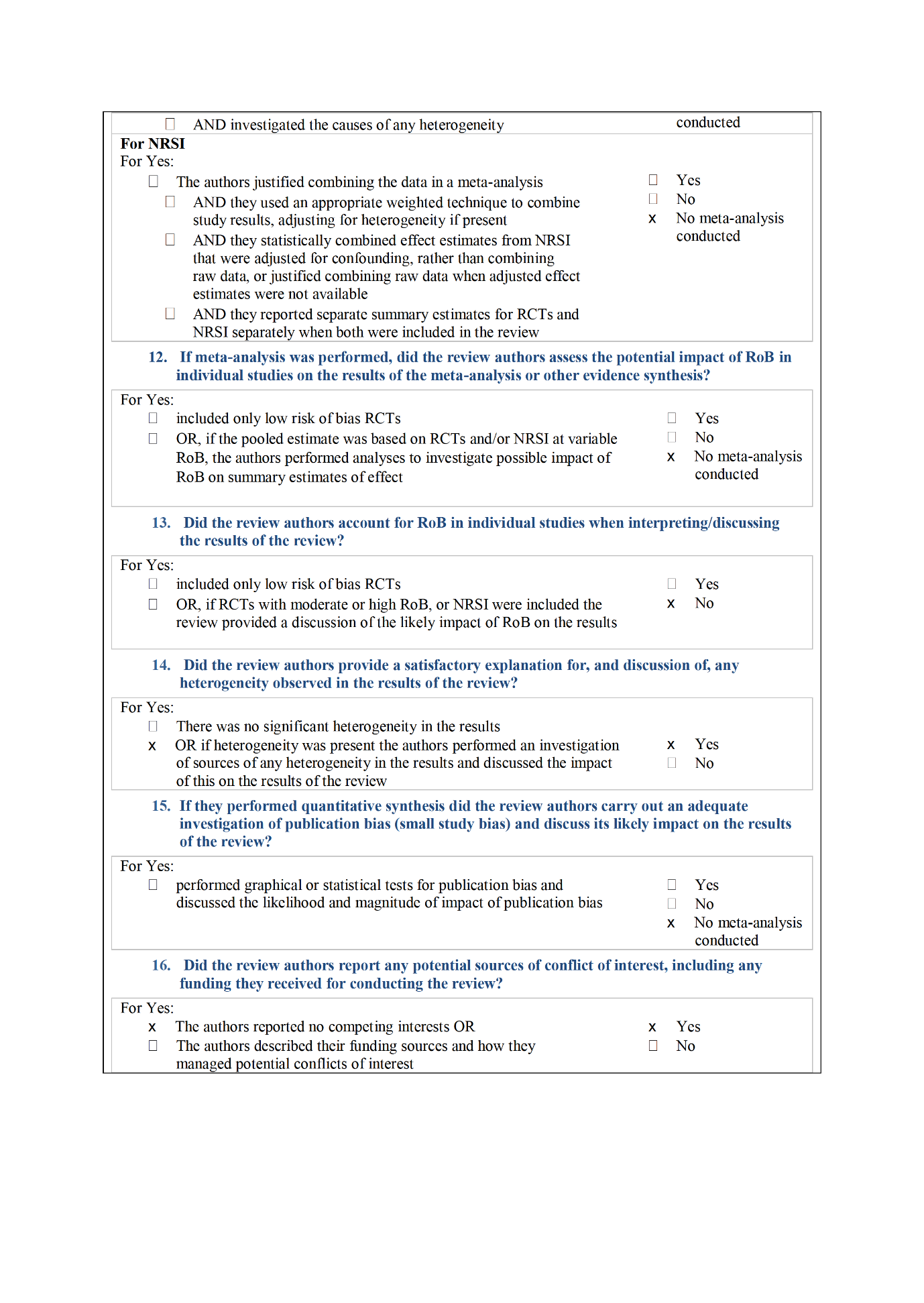


Supplementary Figure 2. Assessment of narrative reviews according to SANRA criteria.

Shackley and Clarke. 2005


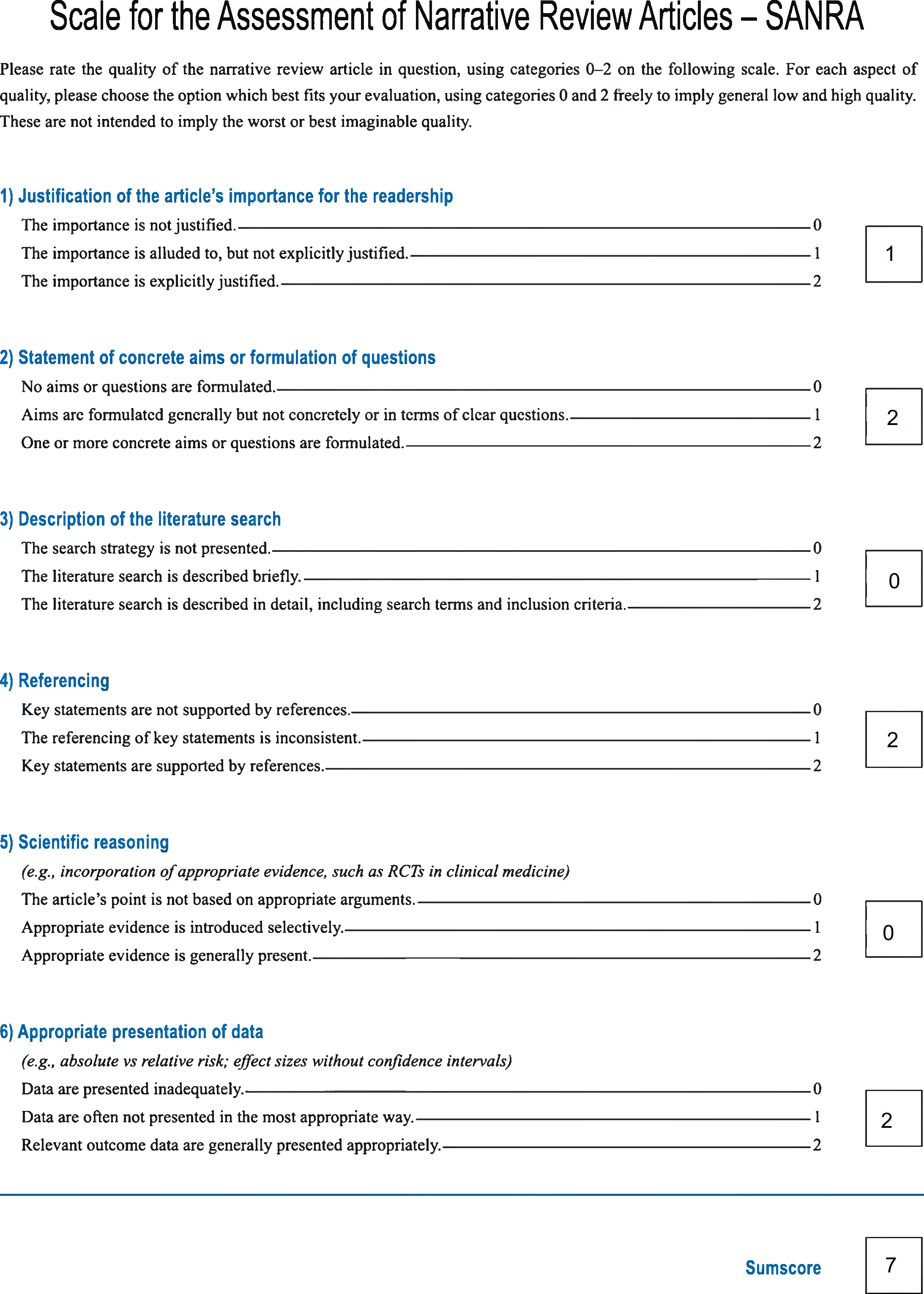


Jacobs. 2012


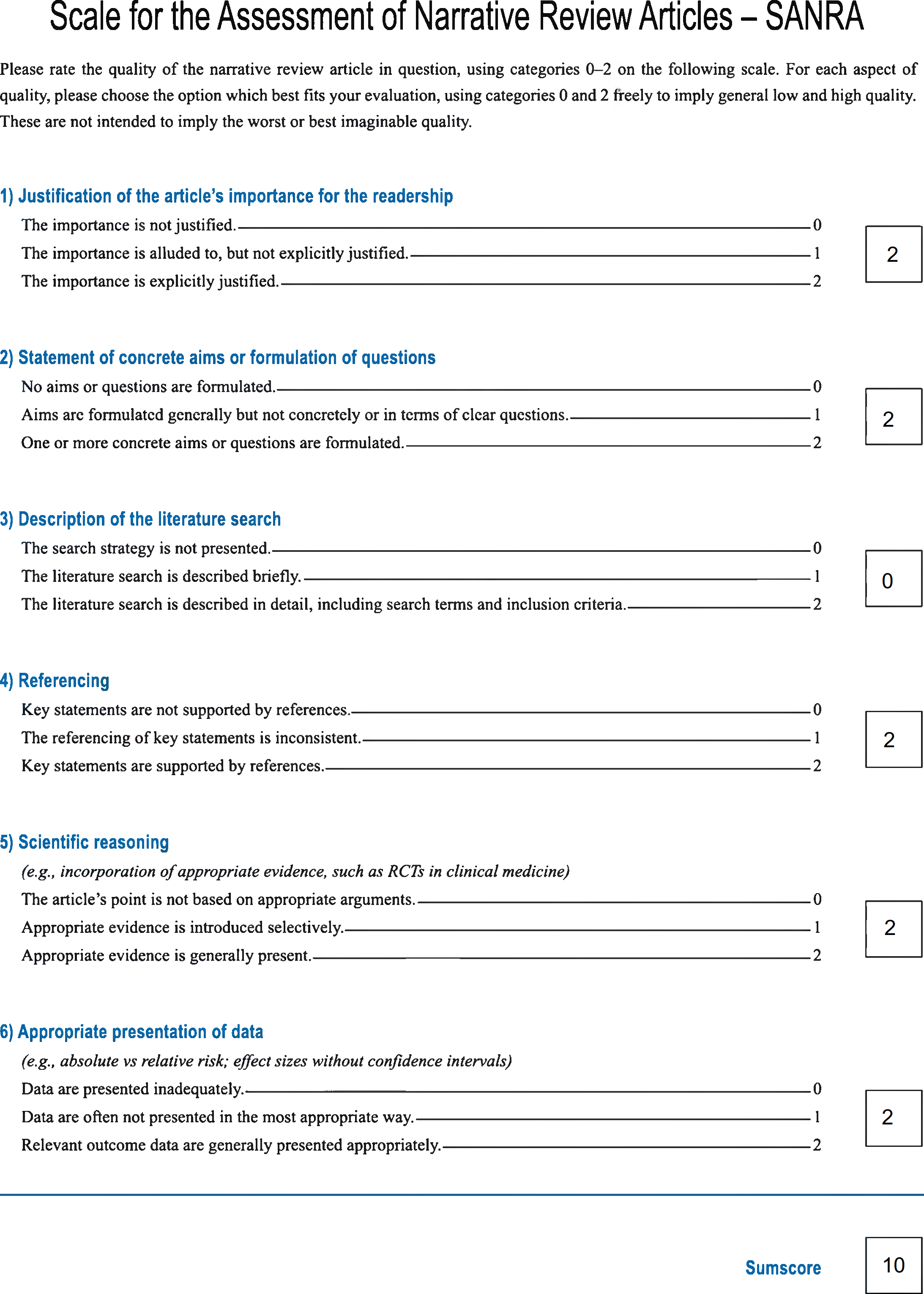

Supplement: Supplementary file 1 — Supplementary Material [file BCO2-2-140-s001.docx]
